# Pancreatic Cancer (PDAC): Introduction of Evidence-Based Complementary Measures into Integrative Clinical Management

**DOI:** 10.3390/cancers12113096

**Published:** 2020-10-23

**Authors:** Valerie Jentzsch, James A. A. Davis, Mustafa B. A. Djamgoz

**Affiliations:** 1 Department of Life Sciences, Neuroscience Solutions to Cancer Research Group, Imperial College London, South Kensington Campus, London SW7 2AZ, UK; valerie.jentzsch16@imperial.ac.uk (V.J.); james.davis@fcdo.gov.uk (J.A.A.D.); 2 Business School, Imperial College London, South Kensington Campus, London SW7 2AZ, UK; 3 Biotechnology Research Centre, Cyprus International University, Haspolat, Nicosia, TRNC, Mersin 10, Turkey

**Keywords:** pancreatic cancer, PDAC, gemcitabine, integrated management, scheduling, diet, glycemic index, vitamins, nutraceuticals, lifestyle factors, clinical trial

## Abstract

**Simple Summary:**

Pancreatic ductal adenocarcinoma is a devastating disease that is very hard to treat. Here, we advance and evaluate the notion that the best possible management currently would be possible by combining clinical procedures with evidence-based complementary measures. We evaluate three categories of such complementary measures: Diet (background and specific), nutraceutical agents and lifestyle factors. Altogether, these include alkalinity, low-glycemic index, low-cholesterol, red meat, fish, fruit/vegetables, dairy, honey, coffee, vitamins A, C, D, E, genistein and curcumin (dietary issues); propolis, triptolide and cannabidiol (nutraceuticals); and obesity, diabetes, smoking, alcohol and exercise (lifestyle factors). The available evidence is considered by four criteria: clinical trials, meta-analyses and in vivo and in vitro data. A total of nine agents satisfy these criteria. These are combined and divided into two groups. Finally, a scheme is proposed for integrating the two groups with gemcitabine chemotherapy on a weekly cycle.

**Abstract:**

The most common form of pancreatic cancer is pancreatic ductal adenocarcinoma (PDAC), which comprises some 85% of all cases. Currently, this is the fourth highest cause of cancer mortality worldwide and its incidence is rising steeply. Commonly applied clinical therapies offer limited chance of a lasting cure and the five-year survival rate is one of the lowest of the commonly occurring cancers. This review cultivates the hypothesis that the best management of PDAC would be possible by integrating ‘western’ clinical medicine with evidence-based complementary measures. Protecting the liver, where PDAC frequently first spreads, is also given some consideration. Overall, the complementary measures are divided into three groups: dietary factors, nutraceutical agents and lifestyle. In turn, dietary factors are considered as general conditioners, multi-factorial foodstuffs and specific compounds. The general conditioners are alkalinity, low-glycemic index and low-cholesterol. The multi-factorial foodstuffs comprise red meat, fish, fruit/vegetables, dairy, honey and coffee. The available evidence for the beneficial effects of the specific dietary and nutraceutical agents was considered at four levels (in order of prominence): clinical trials, meta-analyses, in vivo tests and in vitro studies. Thus, 9 specific agents were identified (6 dietary and 3 nutraceutical) as acceptable for integration with gemcitabine chemotherapy, the first-line treatment for pancreatic cancer. The specific dietary agents were the following: Vitamins A, C, D and E, genistein and curcumin. As nutraceutical compounds, propolis, triptolide and cannabidiol were accepted. The 9 complementary agents were sub-grouped into two with reference to the main ‘hallmarks of cancer’. Lifestyle factors covered obesity, diabetes, smoking, alcohol and exercise. An integrative treatment regimen was devised for the management of PDAC patients. This involved combining first-line gemcitabine chemotherapy with the two sub-groups of complementary agents alternately in weekly cycles. The review concludes that integrated management currently offers the best patient outcome. Opportunities to be investigated in the future include emerging modalities, precision medicine, the nerve input to tumors and, importantly, clinical trials.

## 1. Introduction

Pancreatic cancer is currently the fourth highest cause of cancer mortality worldwide with estimates of 55,440 new cases and 44,330 deaths in 2018 [1]. This is primarily an age-related neoplasm with a median age at diagnosis of about 71. With many developed countries facing the prospect of ageing populations, the incidence level is expected to rise over the coming years [2]. Indeed, incidence is predicted to rise steeply in the foreseeable future and pancreatic cancer may become the biggest cause of cancer-related deaths in the USA by 2030 [3].

The most common form of pancreatic cancer is “pancreatic ductal adenocarcinoma” (PDAC) [4]. This is responsible for some 85% of the cases and is the main focus of this review. PDAC arises in the pancreatic ducts, which run through the middle of the organ and perform its main exocrine functions. The liver is often the first major organ to be metastasized due to the proximity of the hepatic portal vein [5]. Since this spread is not long-distance, this is sometimes referred to as “oligometastasis” [6]. 

Most patients with PDAC remain asymptomatic until the cancer reaches an advanced stage. Genetic analysis suggested that almost 19 years can pass between tumor initiation and metastasis [7]. Once the symptoms emerge, they are often non-specific and variable, with pain in epigastrium and back reported in 70–80% of cases. Other symptoms include lack of appetite and unexplained weight loss. In particular, jaundice can be indicative of PDAC either because the tumor is located at the head of the pancreas (thereby obstructing the bile duct) and/or metastasis to the liver has occurred [5,8]. In fact, liver failure is frequently the main cause of death from PDAC. In the context of integrated management, therefore, complementary remedies should ideally also protect the liver.

The current lack of efficacious diagnostic techniques hinders efforts to manage the disease. Identifying cysts or abnormal structures within the pancreas through ultrasound, magnetic resonance imaging or positron emission tomography often have insufficient sensitivity and selectivity to enable specific and effective therapy. Molecular characteristics of the disease are currently being elucidated in depth and used for diagnosis and subclassification, based upon novel biomarkers such as microRNAs, carbohydrate antigens and methylation biomarkers [9,10]. By such subclassification of PDAC, it may then be possible to determine which treatment will be most effective for an individual patient [11].

In spite of continuing advances, however, PDAC remains one of the most difficult cancers to treat, with the current five-year mortality rate being only around 6% (2% if the cancer has already metastasized) [12]. The strong metastatic potential of PDAC is due to the presence of micro-metastases at systemic sites even at early stages of the cancer [13]. This raises the possibility, therefore, that ‘integrated management’, combining the experience from clinical medicine with evidence-based complementary measures might give PDAC patients the best chance of survival [14,15,16]. An initial idea for the potential benefit of complementary (dietary and lifestyle) measures can be inferred epidemiologically from the fact that the incidence and mortality rates are much higher in relatively developed countries with rich diets [12]. 

Central to our approach are two philosophies. First, cancer is primarily an epigenetic disease with genes responding significantly to diet and lifestyle [17]. Thus, complementary procedures, including diet, can go a long way towards cancer management and survival [18]. Indeed, it has been estimated that ca. 40% of the common cancers can be prevented by lifestyle, including dietary changes and, thus, are modifiable [19,20]. Importantly, however, cancer has an extremely complex pathophysiology, changing in time and space during its progress and response to treatment. It must therefore be managed dynamically by experts employing evidence-based remedies. Second, we are under the constant influence of pro- and anti-cancer influences during our lives. So, if we can keep their balance with the anti-cancer ‘arm’ stronger, patients can live with cancer, even chronically [21]. With medical science advancing in the current post-genomic era faster than ever before, the extra time gained can enable patients to from emerging new therapies. Some 50% of oncologists indeed are interested in complementary therapies and patients are increasingly using these in their strive to achieve better outcomes [22,23,24]. Accordingly, several major cancer treatment centers around the world include ‘integrated management’ in their clinical programs, for example at Abramson (Penn Medicine), MD Anderson, The Mayo Clinic, Memorial Sloan Kettering Cancer Centre and The Harvard Law School Food Law and Policy Clinic [25]. 

Overall, this topic is gaining in prominence and similar reviews have previously dealt with colorectal cancer [26] and prostate cancer [27], some in relation to associated pathologies such as pain [28]. Other articles have dealt with the use of complementary agents/integrated management of cancer in specific countries, e.g., Australia [29,30], Germany [31] and several Middle Eastern countries, including Israel [23,32].

**Figure 1:**
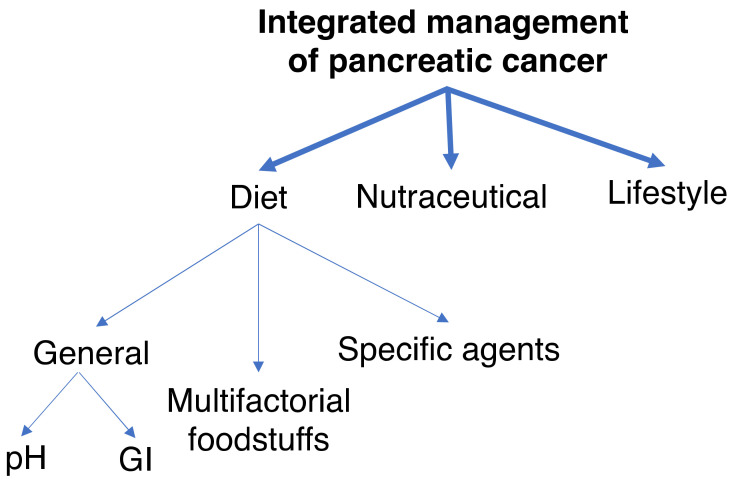
Plan of review aiming at integrated management of pancreatic cancer. Three main complementary approaches are considered—diet, nutraceutical supplements and lifestyle. In turn ‘diet’ is divided into general conditioning factors, mainly acidity (pH) and glycemic index (GI), multifactorial foodstuffs and specific agents (active ingredients).

The current review covers several inter-related topics (Figure 1). First, we give an overview of the current clinical therapies for PDAC and highlight their limitations. The complementary measures are considered mainly in relation to chemotherapy, and include 3 broad areas—diet, nutraceuticals and lifestyle factors. In turn, dietary measures are divided into background conditioners, multi-factorial foodstuffs and specific agents. The effectiveness of such agents in combination with gemcitabine chemotherapy has been evaluated based upon the following: (1) Double-blind clinical trials, (2) meta-analyses, (3) in vivo animal models and (4) in vitro experiments. Here, we only consider agents for which there is consistent evidence from across all four categories. In particular, all the adopted complementary agents have been subject to at least one clinical trial. The relevant past and ongoing clinical trials are summarized in Table 1.

In making the most of the available evidence, we have taken into consideration generation of synergy, avoidance of clash, monitoring of effectiveness as much as possible and taking calculated risk (i.e., accepting positive evidence even when present alongside neutral evidence, but rejecting anything that may include negative evidence). The last point is important especially in later stages of disease. Due to the wide nature of the topics covered, we have included definitions and a glossary of some of the relevant key terminology, also to help non-specialists, including patients.

## 2. Clinical Therapies—An Overview

Surgery is the only potentially curative measure for PDAC and is an evolving field [39,40] Overall, only some 20% of patients are suitable for surgery at the time of diagnosis and 80% of these will suffer a fatal relapse at some point post-surgery [41]. Possibilities for resection are limited by several major blood vessels, nodes of the lymphatic system and vital organs, including the liver, stomach, gallbladder and duodenum located near the pancreas. Tumors are considered ‘operable’ if there is no invasion into the circulatory system and no metastases. Surgery usually entails either (i) a pancreaticoduodenectomy (removal of part of the pancreas, stomach, duodenum and gall bladder) for tumors found at the head of the pancreas or (ii) a pancreatectomy (removal of the pancreas partially or wholly) for those with tumor in the main body or tail [42]. Despite recent improvements in perioperative care, morbidity post-surgery is still around 40%. Resection is often combined with radio- and/or chemotherapy, aiming to eliminate micro-metastases and improve survival in an adjuvant setting [42,43,44]. 

Chemotherapy is an important part of PDAC treatment [45]. It may be applied systemically if the cancer has metastasized or may be combined with surgery in a neo-adjuvant or adjuvant setting [1,46]. Chemotherapy with gemcitabine is the first-line treatment against PDAC and is well tolerated with low side effects [42]. Gemcitabine (Gemzar®) is an ‘antimetabolite’ pro-drug which becomes active once phosphorylated into gemcitabine-P (diphosphate or triphosphate) inside cells. Antimetabolites are cell-cycle-specific. They attack cells at very specific phases in the cycle and work best on fast-dividing cells, hence cancer cells. Once inside cancer cells, incorporation of gemcitabine-P into DNA inhibits its synthesis and thereby cell growth, ultimately leading to cell death. A further mode of action of gemcitabine is production of reactive oxygen species (ROS) that damage DNA and prevent growth [47,48]. Most commonly, gemcitabine is administered in weekly cycles, i.e., drug given on one day followed by 6 days of ‘rest’, and this is continued for several (ca. 7) weeks. Then, depending on progress, treatment may be continued on a modified cycle. Here, we have adopted the common cycle as the basis of the integration.

Despite there being much research into chemotherapy, the survival rate has barely improved over the last ten years. One reason for this is the eventual onset of drug resistance [49]. In advanced PDAC, gemcitabine-based combination chemotherapies can be used. The most commonly used drug cocktail is FOLFIRINOX (combination of folinic acid, fluorouracil, irinotecan and oxaliplatin), which provides a one-year overall survival rate ~28% higher than gemcitabine alone, but its effect on quality of life is worse, including fatigue, diarrhea and sensory neuropathy [50]. Accordingly, FOLFIRINOX is recommended only to patients with a good performance status [42]. In another combination therapy, nab-paclitaxel is added to a gemcitabine regimen, without any increase in toxicity, and this has been shown to extend patient life by two months, i.e., ~32% [51,52]. There is increasing work being done on novel combinations, including sequential chemotherapies, e.g., gemcitabine plus capecitabine or FOLFIRINOX followed by gemcitabine [42,53,54,55]. In addition, combination of chemotherapy and radiotherapy is also currently being trialed post-surgically [https://clinicaltrials.gov/ct2/show/NCT01013649].

We should add that a further difficulty in the chemotherapy of PDAC is the desmoplasia (growth of dense fibrous or connective tissue around the neoplasm) that arises due to the overproduction of extracellular matrix proteins and pancreatic stellate cells (PSCs). Desmoplasia can comprise up to 80% of the tumor volume, compress vasculature and result in increased physical resistance to chemotherapy and radiotherapy [56]. A novel combination involves priming the tumor tissue to alter the microenvironment and thus make it more accessible to treatment. For example, inhibiting rho-kinase with fasudil was shown to increase response to chemotherapy and reduce metastasis in murine models [57]. 

Radiotherapy, most commonly high-energy ionizing X-rays, kills cells by damaging their DNA and triggering oxidative stress (e.g., by ROS production), thereby hindering cell division and causing cell death [58]. Studies on the effectiveness of radiotherapy on PDAC so far have remained inconclusive, with a number of clinical trials producing conflicting results [53]. Nevertheless, the technology of radiotherapy is evolving and its usefulness in treating PDAC is likely to grow [59,60,61]. 

Targeted therapies aim to exploit specific genetic or molecular targets on the tumor cells themselves and/or cells of the stroma. A number of key signaling pathways have been identified as potential targets for therapy, but few have provided significant improvements in patient survival rates [62,63]. In particular, overexpression of epidermal growth factor receptor (EGFR) occurs in >90% of PDACs and is generally associated with poor prognosis. Surprisingly, however, the addition of EGFR-targeted treatment to chemotherapy in patients with locally advanced or metastatic disease did not improve progression-free or overall survival [64]. Any benefit of EGFR inhibitors may be limited to patient subgroups not yet clearly defined, as with the comparable situation in triple-negative breast cancer [65]. Importantly, the efficacy of gemcitabine was shown to increase by combination with inhibition of insulin-like growth factor (IGF) signaling, which would highlight the importance of dietary glycemic index in PDAC management (Section 3.1.2) [66]. Other molecular targets include transforming growth factor-beta (TGFβ), Wnt, Notch and Hedgehog [67]. However, clinical trials with drugs targeting these mechanisms, including in combination with backbone treatments like FOLFIRINOX, have not produced promising results [68]. On the other hand, a phase I trial using the enzyme pegylated hyaluronidase, to digest the stroma, increased the penetration and effectiveness of gemcitabine [69]. 

Finally, immunotherapy is a relatively new but growing and highly promising treatment modality for cancer, including PDAC [70,71]. Drugs blocking immune checkpoints and their receptors, including cytotoxic T-lymphocyte antigen 4 (CTLA4) and programmed death 1 (PD-1), are showing some effectiveness against PDAC, especially in combination with other treatments [72,73,74]. For example, the immune modulator IMM-101 has demonstrated improvements in survival in combination with gemcitabine [73]. However, difficulties with immunotherapies have arisen due to the fact that PDAC is not ‘immunogenic’ and the dense tumor microenvironment suppresses T-cell activity, indicating that multiple pathways may have to be targeted to upregulate the immune response. Furthermore, there are serious issues of safety. Another developing area of immuno-oncology is cancer vaccines, some in combination with chemotherapy [45,75,76]. 

## 3. Dietary Considerations

Although the importance of food in health has been recognized as early as Hippocrates, it has somewhat been ignored over the years and nowadays reference is often made to “Food is the forgotten medicine” [https://collegeofmedicine.org.uk/events/#!event/2016/6/8/food–8211–the–forgotten–medicine]. Consequently, even major cancer charities often do not give specific advice about diet and just say “eat healthily!” Here, we have aspired to evaluate the existing evidence critically to offer specific advice about diet and its combination with clinical treatments.

Pancreas is a hormonal organ (both endocrine and exocrine). As well as producing hormones (mainly insulin), it itself responds to hormones (e.g., estrogen) and growth factors (e.g., EGF) [77]. It would naturally be expected, therefore, that the pancreas and PDAC would be sensitive to the body’s biochemistry and chemical balance, as with other hormone-sensitive cancers. Indeed, not surprisingly, diet and other chemical factors are increasingly being recognized for their importance in PDAC development and progression, even treatment [78,79,80]. Azimi et al. [81] originally identified 12 natural compounds with beneficial effects against PDAC cells through four or more mechanisms. Several of these have been evaluated and found indeed to be inversely associated with PDAC risk [82]. It has even been suggested that diet can have a serious impact at a much earlier stage than previously realized and diet during adolescence and midlife can significantly increase the risk of cancer in later years [83]. Accordingly, there is potential to reduce PDAC incidence by making people more aware of the risks at an earlier stage. In addition, weight loss in PDAC patients receiving chemotherapy is common and associates with worse prognosis. In such situations, intake of supplements has been shown to promote weight gain and improve fatigue and overall quality of life [84].

We should note, however, that foodstuffs are invariably multi-factorial, i.e., contain more than one active ingredient which could generate antagonistic effects [85]. A well-known case is centered around cytochrome P (CYP), an enzyme which can break down chemotherapeutic drugs and reduce their effectiveness [86]. Accordingly, foods that may contain inhibitors of CYP should be avoided or minimized during treatment. These include grapefruit, oranges, pomegranate and vegetables such as cabbage and onion [87]. Asparagus also has been highlighted. Although this is highly alkaline and rich in protein and fiber, it also contains the enzyme *asparagine* which has been shown to facilitate metastasis of breast cancer in an animal model [88]. 

There are several aspects of diet that relate to PDAC. Here, we divide dietary factors into two broad categories: General and specific (Figure 1).

### 3.1. General Dietary Factors

We consider the possible impact upon PDAC of three general characteristics—acidity, glycemic index and cholesterol—that can be associated with any diet.

#### 3.1.1. Acidity

The ‘acidity’ of human blood is highly stable (pH = 7.35–7.45) in healthy individuals and cancer patients [89]. In contrast, the tumor environment is well known to be acidic (pH ~6.5) which promotes the invasiveness of the cancer cells (e.g., by activating proteolytic enzymes and digesting their surroundings) whilst inhibiting the growth of the normal host cells [90,91,92]. A major mechanism controlling the tumor acidity is the sodium-hydrogen exchanger (HNE1), which extrudes acid (hydrogen ions—H^+^) in exchange for sodium (Na^+^). This exchanger is upregulated by oncogenes and growth factors [93]. Indeed, NHE1 is expressed and associates with EGF signaling in PDAC cells in vitro and in vivo, and inhibiting NHE1 with cariporide reduced growth and invasiveness [94]. Another remarkable property of a healthy pancreas is its secretion of a bicarbonate-rich fluid through its ducts to regulate digestion in the stomach [95]. Secretion occurs from the ‘exocrine’ part of the pancreas and this is also where cancer mostly arises. Bicarbonate is a major controller of acidity through the action of the enzyme carbonic anhydrase (CA9). This is also upregulated in PDAC and would enhance the extracellular acidification [91,96]. 

Given that an acid environment would promote cancer invasiveness, an alkaline diet might counteract this effect and improve the patient’s prognosis. However, a question that is often asked is whether the pH of food can ultimately influence the acidity of the tumor. A firm answer to such a question would not be easy as it would require measuring pH within the tumor microenvironment and such direct correlative studies have not been carried out. Nevertheless, an insight can be gained from the following consideration: potassium chloride injected into blood is well known to be lethal. This is despite the body possessing several layers of transport mechanism and barriers regulating the level of potassium in tissues. It would be expected, therefore, that protons, another small particle, could similarly enter body fluids and accumulate in the tumor microenvironment to influence the extracellular pH even if to a limited extent. The same would be true for other small molecules including dietary metabolites. In fact, the impact of blood chemical composition on the tumor microenvironment could be greater than expected due to the vascular nature of cancer tissue. In addition, the existence of narrow spaces within the tumor microenvironment coupled with metabolic potentiation could cause significant shifts in the extracellular pH of tumor cells, analogous to the microenvironment of the brain [97]. Indeed, an epidemiological study reported that high fruit and vegetable consumption and low meat intake could significantly alkalinize the urine pH in healthy men and women of ages up to 79 years [98]. Thus, the regime adopted significantly both alkalinized the average pH values by about 0.2 units and increased the blood vitamin C level by some 10%.

**Figure 2:**
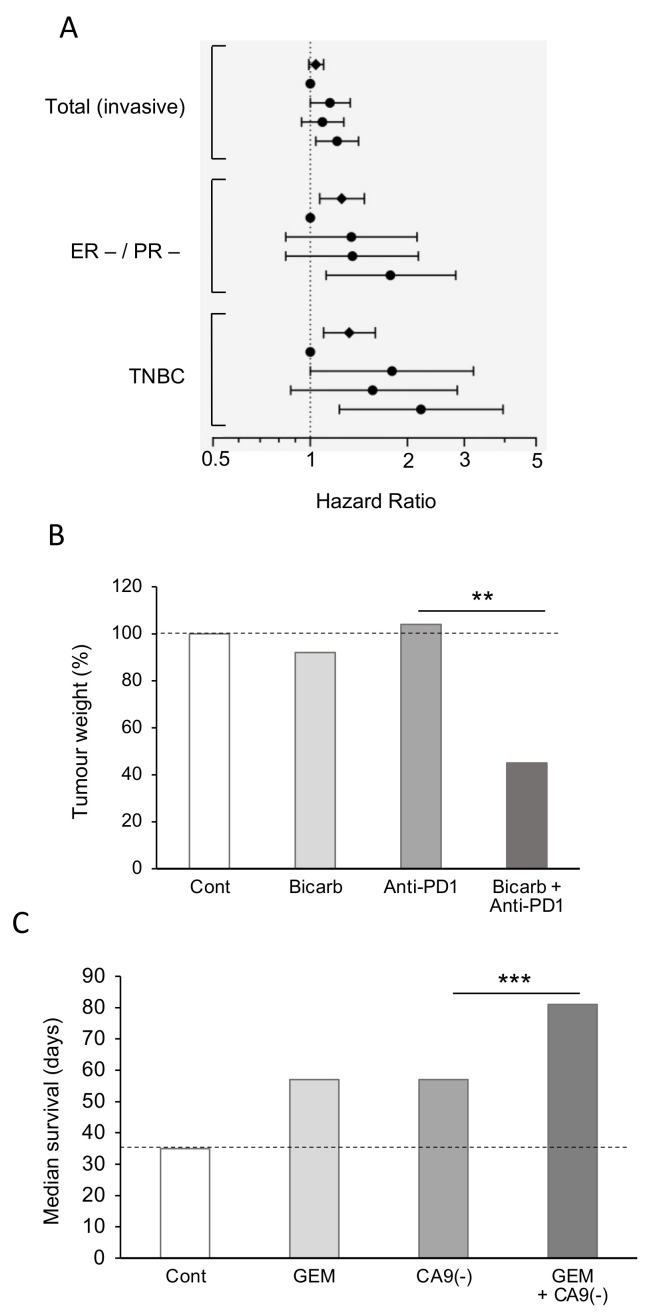
Effects of acidity on cancer. (**A**). Hazard ratios for the potential impact of renal acid load on risk of breast cancer, divided into total (invasive), ER-/PR- and TNBC. ER, estrogen receptor; PR, progesterone receptor; TNBC, triple-negative breast cancer. The dotted vertical line (hazard ratio = 1) denotes no risk. There was a clear risk of acidity on all the different sub-types of breast cancer studied (*p* < 0.05 for total). Modified from Park et al. [99], where further details and primary data can be found. (**B**) Improvement of immunotherapy effectiveness in mice bearing Panc02 pancreatic tumors. The measured tumor weights are plotted relative to the control level (100%). Mice were treated with a combination of anti-PD1 antibodies with or without bicarbonate. Control (Cont) was tap water, value marked by the horizontal dotted line for ease of comparison. Significant reduction in tumor weight was observed only by combining anti-PD1 treatment with bicarbonate (*p* < 0.005) (**). Data replotted from Pilon-Thomas et al. [92], where further details and primary data can be found. (**C**) Survival data (median values) for mice with PK-8 pancreatic cancer xenografts. Treatments were as follows: untreated control (Cont), treated with gemcitabine (GEM), CA9-depleted but untreated (CA9(−)) and CA9-depleted treated with gemcitabine (CA9(−) + GEM). Control value is marked by the horizontal dotted line for ease of comparison. Data obtained from the Kaplan–Meier analyses of McDonald et al. [100], where further details can be found. Compared with the control, the effect of CA9 depletion (i.e., alkalinization) was significant (*p* < 0.01). Gemcitabine treatment on CA9-depleted mice was significantly more effective than CA9-depletion alone (*p* < 0.001) (***). Since the data for CA9-depletion and gemcitabine alone were very close, it can also be inferred that the effect of gemcitabine on CA9-depleted mice would be equally significant, compared with gemcitabine alone (not shown).

In spite of our considerable knowledge of tumor acidity, there have not been many systematic studies linking acidity of diet to PDAC. However, there is evidence from other cancers. For example, in a meta-analysis, Park et al. [99] showed that diet-dependent acid load was associated positively with risk of breast cancer, for all sub-types studied (Figure 2A). In a pre-clinical mouse study, also, Pilon-Thomas et al. [92] demonstrated (i) that bicarbonate (‘alkaline’) water could suppress growth of some melanoma cells and (ii) that it could potentiate the effectiveness of immunotherapy against an induced pancreatic tumor (Figure 2B).

As noted above, one of the mechanisms that regulates the pH of the body is the enzyme CA9. The activity of CA9 acidifies body fluids, so inhibiting it causes alkalinization [96]. Consistent with this, McDonald et al. [100] showed in a mouse model that CA9-knockout increased median survival by ca. 60%. Importantly, in CA9-knockout mice with PDAC xenografts (i) tumor burden and metastases were reduced and (ii) the effectiveness of gemcitabine in prolonging survival was potentiated, the median increasing by ca. 40% (Figure 2C). These effects were significant and presumed to be due to ‘background’ alkalinization [100]. 

Importantly, a recent study reported the retrospective effects of combining an alkalinizing diet with chemotherapy (including gemcitabine) on 28 Japanese patients with advanced or recurrent PDAC [101]. The diet was based on at least 400 g of fruits and vegetables per day and no meat or dairy products. In addition, daily, all patients received supplementary intravenous (i.v.) vitamin C (25–50 g/day) and some were also given oral bicarbonate (3.0–5.0 g/day) when their urine pH did not increase above 7.0 or when patients wished to take it. This study revealed two significant results. First, the applied alkalinization regime increased the average urine pH from 6.39 to 6.85. This confirmed that diet can affect the pH of body fluids of PDAC patients as in healthy individuals [98]. Second, the average median overall survival was increased from 4.7 to 16.1 months for patients with urine pH levels of <7.0 and >7.0, respectively (time to death increasing from ca. 18 months to >50 months) [101]. Both effects were statistically significant. Interestingly, this study would also reinforce the supplementary benefits of i.v. vitamin C and avoidance of dairy.

In conclusion, an alkaline diet can provide benefits for patients of PDAC (and other cancers) both normally and during chemotherapy. There is certainly no evidence to the contrary. In contrast, fizzy drinks, dairy and red meat are acidic and should be avoided. Alkalinity might not only reduce cancer risk and associated symptoms such as weight loss and constipation, it may also counteract risk factors of the disease including diabetes and obesity, as well as improve general health [102,103]. Finally, first, since urine pH appears to relate to dietary acid–base load, it could be used conveniently by individuals as a monitor of the impact of diet [98,101]. Second, there is some evidence that resistance to gemcitabine may also be reduced in an alkaline environment and this can be exploited by designing special biomaterials [104,105]. Furthermore, benefits could be gained by combining chemotherapy with pH-modulating drugs [106]. Third, since the kidney is the body’s main pH regulator, it would be essential to ensure a healthy renal function. Accordingly, patients may benefit from checking the pH of their urine (first of the day, using basic Litmus paper) as a means of monitoring the impact of their diet on the body’s acidity. Fourth, it is well known that cancer patients undergoing chemotherapy frequently suffer from vomiting. Although this is highly unpleasant, since vomit is very acidic, it could be beneficial in the context of pH regulation by the body being driven alkaline, at least transiently.

#### 3.1.2. Glycemic Index

**Figure 3:**
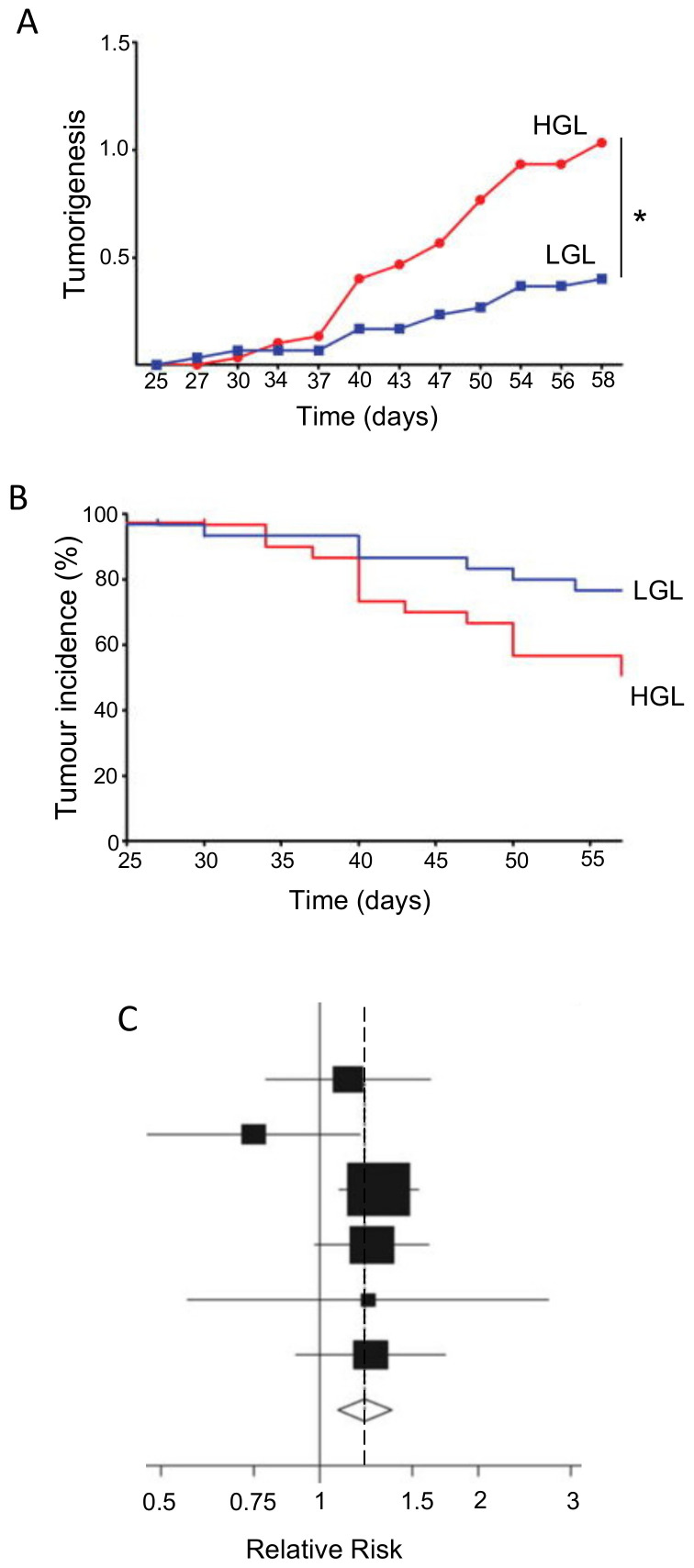
Effects of glycemic load on tumorigenesis. (**A**,**B**) Effects of diet with high or low glycemic load (HGL and LGL, respectively) on a rat carcinogenic model of breast cancer. (**A**) Effect on tumorigenesis, evaluated as the number of palpable tumors for each rat. LGL versus HGL was associated with a reduction in tumorigenesis by some 60% at the end of the experiment (*p* = 0.03). Time is from the start of carcinogenic treatment. (**B**) Data showing effect of glycemic load on tumor incidence (quantified as the percentage of cancer-free rats). Final cancer incidence was reduced by some 50% in LGL versus HGL (*p* = 0.03) (*). Time is from the start of carcinogenic treatment. (**C**) Meta-analysis of pancreatic cancer risk associated with intake of fructose. The vertical line (relative risk = 1) denotes no risk. Diamond indicates the average and the spread of the data (vertical dotted line denotes the average position). In each case, (i) the size of the square is a measure of the ‘weight’ of the study (defined as the inverse of the variance) and (ii) horizontal lines represent confidence intervals. (**A**,**B**) modified from Thompson et al. [108]; (**C**) modified from Aune et al. [112], where further details can be found.

Glycemic index (GI) is a property of food that determines the body’s hormonal response, mainly release of insulin. Glycemic load, which factors in the serving size of a food, is also used. Foods with higher GI, such as refined sugar, tend to need more insulin release for digestion, which could impact upon PDAC directly and/or indirectly. Given the association of diabetes and obesity with PDAC, diets with a high GI may promote disease emergence and progression. This hypothesis was supported by animal experiments which showed that high-GI diets increased the risk of type II diabetes, a well-known risk factor for PDAC [107]. Similarly, in humans, diets of low-GI were found to have a protective effect against breast cancer, suppressing tumorigenesis and prolonging disease-free period (Figure 3A,B) [108]. Meta-analyses of various cancers, including PDAC, have produced mixed but promising results [109,110,111]. Low-GI diets could reduce the risk of PDAC, depending on the contents of the diet (Figure 3C) [112]. Specifically, low-GI diets have been shown to improve blood glucose control and could also benefit insulin sensitivity [113]. Furthermore, PDAC can lead to damage of the tissues (β-cells) involved in insulin release, and a high-GI diet could enhance this by ‘positive feedback’, ultimately leading to glucose intolerance [114]. Fizzy drinks are notoriously high in sugar and, consequently, have a high GI. One study showed that drinking two or more such drinks per week increased the risk of developing PDAC. The proposed mechanism behind this association was again linked to high insulin levels, which could promote the proliferation of PDAC cells [115]. In relation to ‘integration’, a recent study suggested that downregulating the glucose transporter GLUT1 can overcome chemotherapy resistance, consistent with a low glucose/GI diet being beneficial during treatment [116]. 

In conclusion, low-GI diets could both prevent and slow the development of PDAC. Importantly, no adverse effect was reported.

#### 3.1.3. Cholesterol

**Figure 4:**
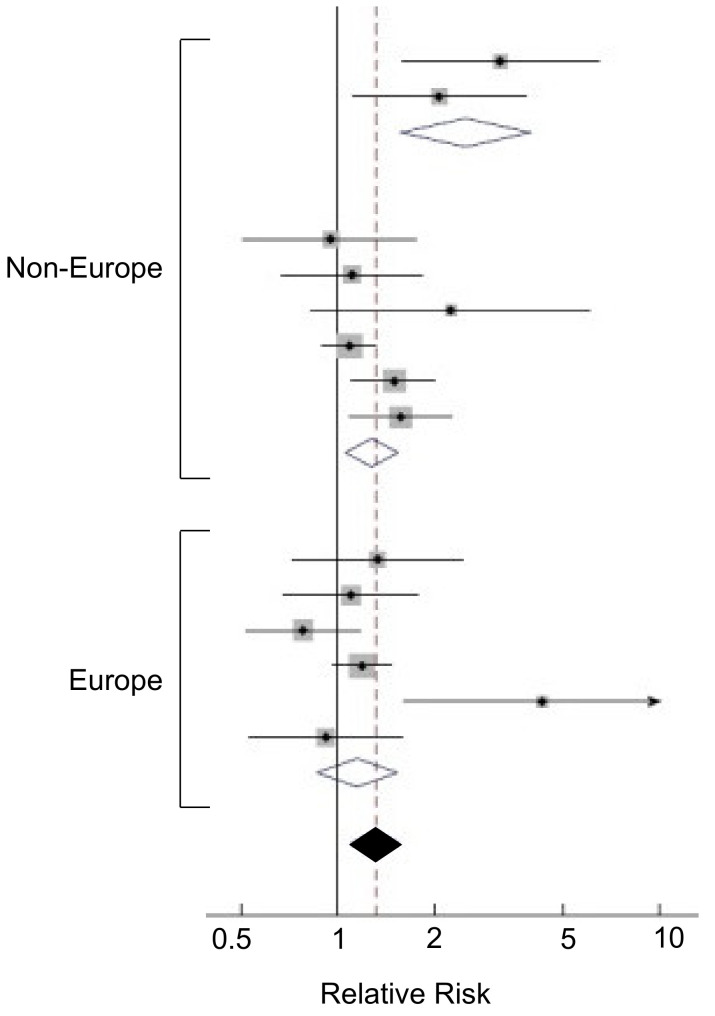
Effects of cholesterol on pancreatic cancer. Forest plot of the effects of dietary cholesterol on pancreatic cancer risk, comparing European and non-European countries (including North America, Australia and Japan). A positive association was apparent in both cases but reached significance only for the latter. Diamonds indicate the average and the spread of the data from individual studies. The black diamond marks the overall values (emphasized by the dotted vertical line). Modified from Wang et al. [118], where further details and primary data can be found.

High levels of cholesterol in diet may also contribute to PDAC but this may vary with geographical location. An initial Canadian study found such a positive association [117]. This was later confirmed for worldwide populations overall, and although the same trend was seen for Europeans, this was not significant (Figure 4) [118]. The exact mechanism(s) of the apparent PDAC–cholesterol association has yet to be elucidated. Directly, excess cholesterol may lead to increased levels of pro-inflammatory cytokines [119]. Increased inflammation could lead to pancreatitis, which is a well-established risk factor for PDAC. Also, the build-up of cholesterol may impede bile secretion, leading to reflux in the head of the pancreas where most tumors occur [120]. Alternatively, the impact may be indirect, via cardiovascular effects, including blood pressure [121]. Total serum cholesterol has been suggested as a diagnostic marker for PDAC, due to the increased risk of PDAC associated with higher levels of serum cholesterol [122]. In a recent, detailed study, Oni et al. [123] identified sterol O-acyltransferase 1 (SOAT1) as a key player in sustaining the mevalonate pathway by converting cholesterol to inert cholesterol esters, thereby preventing the negative feedback caused by unesterified cholesterol. It was concluded that inhibiting SOAT1 could be of therapeutic benefit to PDAC. Another aspect of cholesterol control is use of statins, which have been reported to have mixed effects on cancer [124]. As regards PDAC, however, evidence, including a meta-analysis, suggests that use of statins could reduce the risk of the disease [125,126,127]. As regards chemotherapy, one study showed (i) that gemcitabine-resistant PDAC cell lines had higher levels of esterized cholesterol and (ii) that inhibiting cholesterol esterification, which could lead to reduced blood cholesterol, would potentiate the effect of gemcitabine on PDAC in vitro and in vivo [128]. From a practical point of view, patients should maintain low serum cholesterol (which can be easily tested at a low cost).

### 3.2. Multifactorial Foodstuffs

Here, we discuss a number of multifactorial foodstuffs mainly in a preventative, supportive setting. Wherever possible, the evidence is supported by meta-analyses and/or mechanistic considerations.

#### 3.2.1. Red Meat

**Figure 5:**
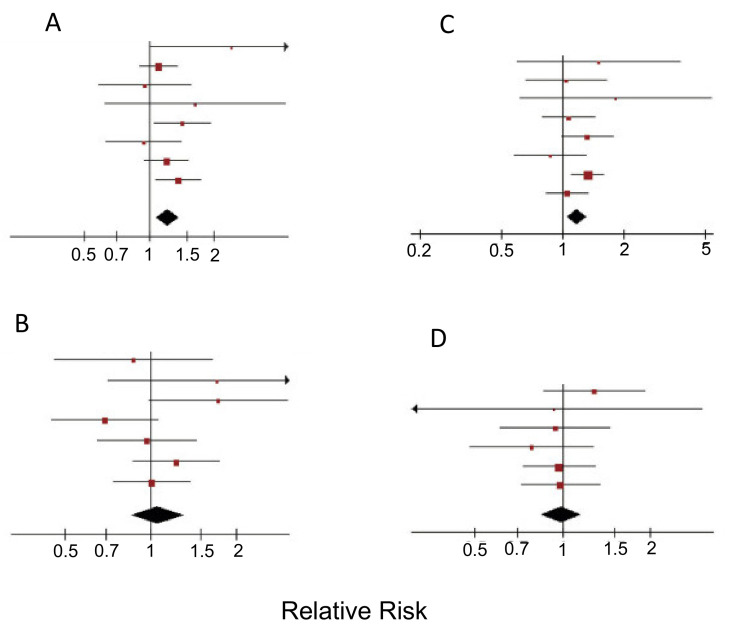
Effects of red meat and processed meat consumption on pancreatic cancer risk. (**A**,**B**) Red meat consumption and pancreatic cancer risk in men (**A**) and women (**B**). Positive relationship was found in men (*p* < 0.01) but not in women (*p* = 0.61). (**C**,**D**) Processed meat consumption and pancreatic cancer risk in men (**C**) and women (**D**). Positive relationship was found in men (*p* < 0.01) but not in women (*p* = 0.88). Diamonds indicate the average and the spread of the data. Modified from Zhao et al. [134], where further details and primary data can be found.

Overall, for processed and unprocessed red meat consumption in the USA, an increase of at least half a serving per day was associated with a 13% higher mortality risk [129]. Indeed, although red meat provides important proteins and micronutrients, consuming large amounts (judged by the UN to be 200 g or more per day) has long been thought to be associated with increased risk of cancer, including PDAC [130,131]. An initial cohort study found that eating red and processed meats increased PDAC risk by some 50% and this was due to the meat itself rather than its preparation [132]. Indeed, a meta-analysis of 800 studies showed a positive association between eating both red and processed meat with developing PDAC [133]. This has been confirmed by a further meta-analysis which showed additionally that men, rather than women, were affected significantly (Figure 5A–D) [134]. In this regard, inherent to red meat is its acidity (pH as low as 5.2). More recent findings suggested that there might also be a hormonal component (consistent with the gender difference) resulting in decreased insulin sensitivity and subsequently an increased risk of diabetes, one of the main risk factors for PDAC [134]. Thus, any red meat consumed should ideally be lean, best from grass-fed animals. Regarding cookery, preparation at high temperatures can introduce carcinogens such as heterocyclic aromatic amines. In addition, curing and preservative processes may generate harmful chemicals including polycyclic aromatic hydrocarbons [135,136]. 

In conclusion, PDAC patients, and those at high risk, should consider replacing red meat with alternative sources of protein such as poultry, fish, legumes and soy products. This may not only reduce the risks associated with red meat consumption but has also been shown to lower cholesterol levels [137]. In addition, avoiding red meat could be beneficial to liver function [138]. 

#### 3.2.2. Fish

**Figure 6:**
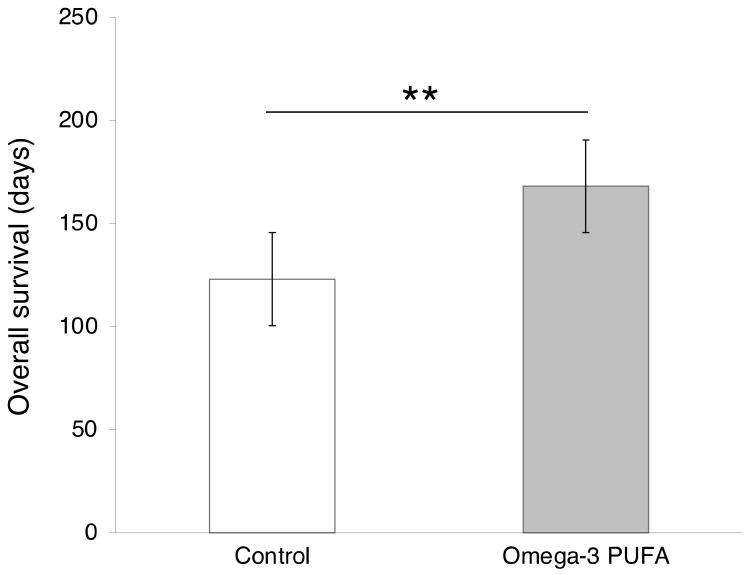
Association of omega-3 poly-unsaturated fatty acid (PUFA) consumption with pancreatic cancer. There was significant improvement in the overall survival of pancreatic cancer patients resulting from omega-3 PUFA supplementation (*p* < 0.01) (**). Histobars indicate means and standard errors (*n* = 527 patients in total). Figure plotted from data given by Ma et al. [141], where further details and primary data can be found.

Fish, particularly oily fish like mackerel, herring and salmon, are excellent sources of omega-3 fatty acids [139]. A decrease in the risk of PDAC has been suggested in relation to an increased consumption of non-fried fish [140]. A systematic, detailed analysis of clinical data concluded that consuming omega-3 fish oils prolonged the survival of PDAC patients with unresectable tumors significantly by some 37% (Figure 6) [141]. Omega-3 fatty acids are anti-inflammatory, anti-proliferative and anti-metastatic [142]. Furthermore, supplementation with fatty acids can improve performance following surgery [139,143]. Additionally, a significant proportion of PDAC patients suffer from cachexia, a serious wasting condition that can accompany late-stage PDAC [144]. Supplementation with fish oils has also been shown to stabilize the weight of PDAC patients, likely through a decrease in the inflammation associated with cachexia. Positive, but not statistically significant, changes in quality of life and a liver protective effect were also reported [144]. Omega-3 poly-unsaturated fatty acids, especially docosahexaenoic acid, accumulate in pancreatic tissues and function through multiple pathways [140,145]. For example, by reducing β-catenin expression, inducing caspase-dependent cell death and decreasing the phosphorylation of Akt (protein kinase B) [145,146,147]. In turn, decreasing the phosphorylation of Akt may improve the effectiveness of chemotherapy by decreasing desmoplasia [146]. 

In conclusion, omega-3 fatty acids can be recommended for PDAC patients and those at risk. Although supplementation does not result in adverse effects, consumption of fish as a whole may provide even greater benefit [139,141]. We should add that considerable work has been done combining gemcitabine chemotherapy with omega-3 supplementation, including i.v., and generally beneficial effects have been obtained [148,149]. A recent clinical trial evaluated this combination on advanced PDAC patients (Table 1, A). It was concluded that anti-cancer effects resulted through immune mechanisms, and a phase III trial was recommended [33]. 

#### 3.2.3. Fruit and Vegetables

It is generally accepted that Mediterranean diet, rich in fresh fruit and vegetables, is the best anti-cancer diet [150,151]. Rather surprisingly, however, two analyses based on European populations concluded that adherence to Mediterranean diet was not associated with PDAC risk [152,153]. Possible anti-cancer roles of fruits and vegetables were recently discussed, and it was suggested that specific components of these might have protective effects [154]. Indeed, apart from their alkalinity, fruit and vegetables, especially dark green leafy vegetables, are rich in carotenes. The latter are fat-soluble photosynthetic pigments colored orange, yellow or red (hence the notion of eating five different colored foods every day). In addition to their role as precursors of vitamin A (Section 3.3.1), carotenes are antioxidant nutrients. Effects of the specific agents found in fruit and vegetables are detailed in the following sections, where further conclusions may be drawn.

#### 3.2.4. Dairy

The possible role of milk and dairy products in cancer including PDAC is controversial and conclusions of meta-analyses have been inconsistent. Some studies found a positive association between dairy consumption and PDAC risk [155]. Other studies, some at specific locations, did not find any association [156,157,158,159]. Some early reports linked the kinds of fatty acids found in dairy products to increased cancer risk. Monounsaturated and saturated fats, including those in dairy (and red meat), were found to increase the risk of PDAC [160]. The stimulation of the duodenum to release the fat-digesting hormone cholecystokinin was thought to increase enzyme secretion from the pancreas, making it more vulnerable to hypertrophy and hyperplasia, as well as possible carcinogens [161]. Another study linked milk consumption with obesity, one of the risk factors for PDAC. One major reason for the apparent inconsistencies in the available data is likely to be the quality of the milk and dairy products in question, dependent on the farming practices involved. A recent study centered on several regions of the USA reported that currently used antibiotics and pesticides were undetectable in organic milk but prevalent in samples of conventionally produced milk [162]. Also, levels of bovine growth hormone and IGF were higher in conventional milk consistent with the presence of synthetic growth hormone [162]. 

There could be two basic reasons for the possible pro-PDAC effects of dairy. First, various growth hormones/factors, including IGF-1, can be present in milk and circulate around the body in cancer patients [163,164,165]. In turn, IGF-1 can promote PDAC growth through a range of mechanisms [166]. Second, milk constituents (e.g., fats and amino acids) can stimulate further secretion of growth hormones/factors, including IGF-1 itself [167]. Importantly, also, daily consumers of milk and other dairy products were over five times more likely to have a *KRAS*-mutated PDAC than non-daily consumers [168]. Oncogenic *KRAS* mutations are the major drivers of PDAC, leading to sustained activation of various intracellular signaling pathways and transcription factors promoting cell proliferation, migration, transformation and survival [169]. 

In conclusion, therefore, in line with our adopted strategic philosophy, we would advocate not taking unnecessary risk and avoiding milk and dairy products. Any milk consumed should be organic and free from hormones and growth factors.

#### 3.2.5. Honey

Honey and some of its constituent polyphenols and byproducts have been shown to have anti-cancer effects through several different mechanisms, such as cell cycle arrest, induction of apoptosis, including that of cancer stem cells (CSCs), and inhibition of cell migration [170,171,172]. Most of the available data come from breast and colorectal cancers [171,173]. Honey may also improve the activity of some chemotherapeutic drugs, but this is yet to be tested on PDAC [174]. A question that is often asked about honey is the potential impact of its sugar content. First, we should stress that honey that may have been adulterated with sucrose or fructose syrup should be avoided. So, it is best to obtain ‘organic’ honey directly from a known local beekeeper. Second, we presume that the sugar element can be outweighed by the anti-cancer agents present in honey. Such agents include chrysin, quercetin, apigenin and caffeic acid phenethyl ester (see Section 4.1 on propolis).

In conclusion, although honey has potential anti-PDAC benefits, its consumption should best be moderated. Nevertheless, for those patients with an irresistible ‘sweet tooth’, an occasional small spoonful of honey, especially Manuka honey, would be acceptable and may even potentiate the effectiveness of chemotherapy [175,176]. 

#### 3.2.6. Coffee

The evidence for the impact of coffee consumption on PDAC is rather mixed. A positive association was reported initially from a small study [177]. A further prospective study found no association with coffee, including total, caffeinated or decaffeinated coffee consumption, especially amongst women [178,179]. In contrast, bigger meta-analyses suggested that there is an inverse relationship between coffee drinking and risk of PDAC [180,181]. Importantly, drinking caffeinated and, to a lesser extent, decaffeinated coffee, was found to be significantly associated with a reduced risk of liver cancer, including pre-existing disease [182,183]. Indeed, a meta-analysis of 12 epidemiological studies suggested that increased coffee consumption can significantly reduce the risk of liver cancer as well as chronic liver disease [184]. A possible mechanism for the anti-cancer effects of coffee includes the presence of antioxidants that can decrease the damaging influence of ROS and inflammation [181]. There is also emerging evidence that caffeine could synergize with gemcitabine chemotherapy on PDAC cells and overcome drug resistance through a novel receptor mechanism, but this area needs much more work before a firm recommendation can be made [185]. 

In conclusion, PDAC patients could benefit, directly and indirectly, from drinking coffee. A useful guide would be 3 cups per day, inferred from the study by Bravi et al. [184]. 

### 3.3. Specific Dietary Agents

A range of natural, especially phytochemical, agents have been highlighted for their effectiveness against pancreatic cancer and its major risk factor, chronic pancreatitis [186]. Here, two main types of specific complementary agent for integration with gemcitabine chemotherapy are considered: nutritional (this section) and nutraceutical (Section 4). The former would be consumed either in normal diet or, if body levels are low, taken as supplements. All the adopted agents have been evaluated in relation to the four levels of evidence outlined in the Introduction. As regards clinical trials, even phase I data have been accepted based upon the fact that these will have been justified and have produced positive or at least neutral (but not negative) outcome. In addition, some are supported by meta-analyses. Experimentally, consistent data are available from combination treatments in vivo and in vitro. Thus, we have adopted 6 dietary and 3 nutraceutical agents.

For all the specific dietary agents we have considered, the known active ingredients, their chemical formulae, main modes of action and natural sources are listed in Table 2.

#### 3.3.1. Vitamin A

**Figure 7:**
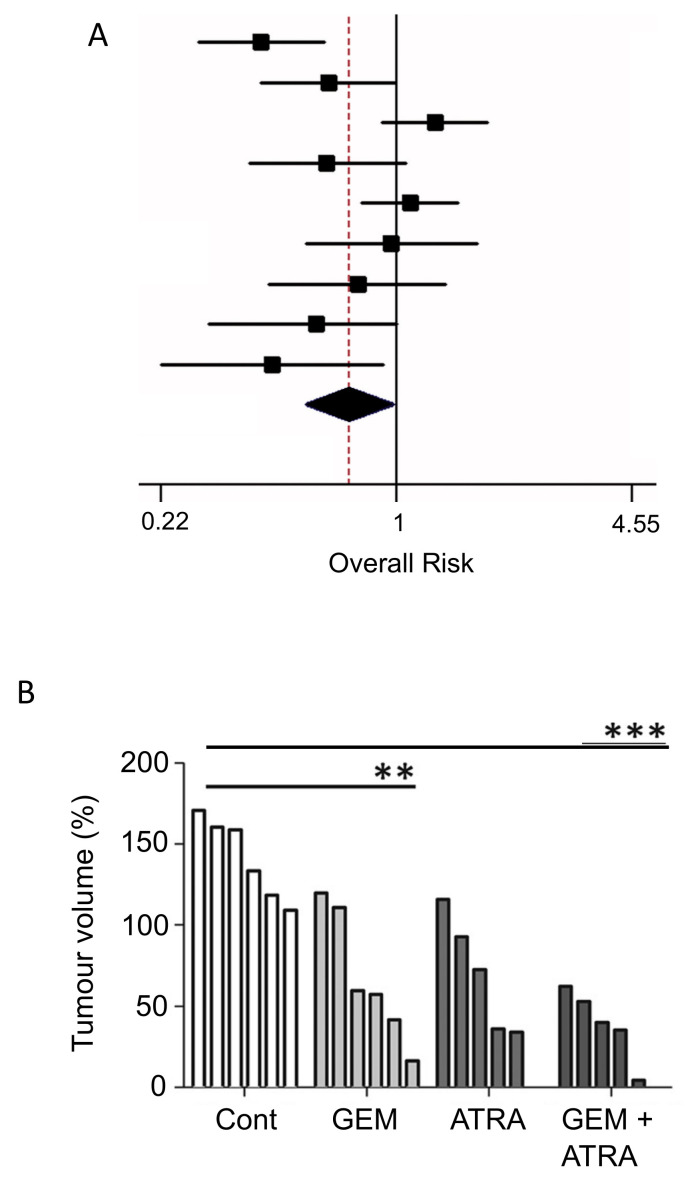
Effects of combining chemotherapy with a dietary compound: Vitamin A. (**A**) Meta-analysis showing significant negative association between β-carotene intake and pancreatic cancer risk (no *p*-value specified). Diamond indicates the average and the spread of the data. Modified from Chen et al. [82], where further details and primary data can be found. (**B**) Effects of gemcitabine (GEM) and ‘all-trans retinoic acid’ (ATRA), alone or in combination, on tumor growth in a mouse KPC model of pancreatic cancer. Each histobar denotes result from a given mouse. The effect of the GEM + ATRA combination was more pronounced than gemcitabine alone (*p* < 0.001/*** and < 0.01/**, respectively). The apparent lack of significance between the effects of the combination and gemcitabine alone is likely to be due to the limited number of mice in each group. Modified from Carapuça et al. [194].

Studies, including meta-analyses, on vitamin A and its precursors (e.g., β-carotene), showed a significant negative association with PDAC risk, with consumption improving patient outcome (Figure 7A) [82,187,188]. A form of vitamin A, all-trans retinoic acid (ATRA), restored quiescence in PSCs through the activation of a retinoic acid receptor, leading to a reduction in desmoplasia in the tumor microenvironment [189]. In combination with gemcitabine, retinoic acid applied to human PDAC cells produced a synergistic effect which could involve (i) reduced expression of CSC markers and (ii) promotion of apoptosis [190,191,192]. Further in vitro experiments on gemcitabine-resistant PDAC cells demonstrated that ATRA was able to enhance chemosensitivity [193]. Use of the mouse in vivo KPC model of PDAC revealed that combination of ATRA and gemcitabine slowed tumor growth more significantly than gemcitabine alone (Figure 7B) [194]. A phase 1B clinical trial has been underway to determine the safety profile of combining ATRA with gemcitabine and nab-paclitaxel (Table 1, B).

In conclusion, vitamin A can be considered for integration with gemcitabine against PDAC. Most people can obtain sufficient vitamin A from the kinds of foods listed in Table 2. If necessary, by medical advice, it can also be taken as a supplement.

#### 3.3.2. Vitamin C

**Figure 8:**
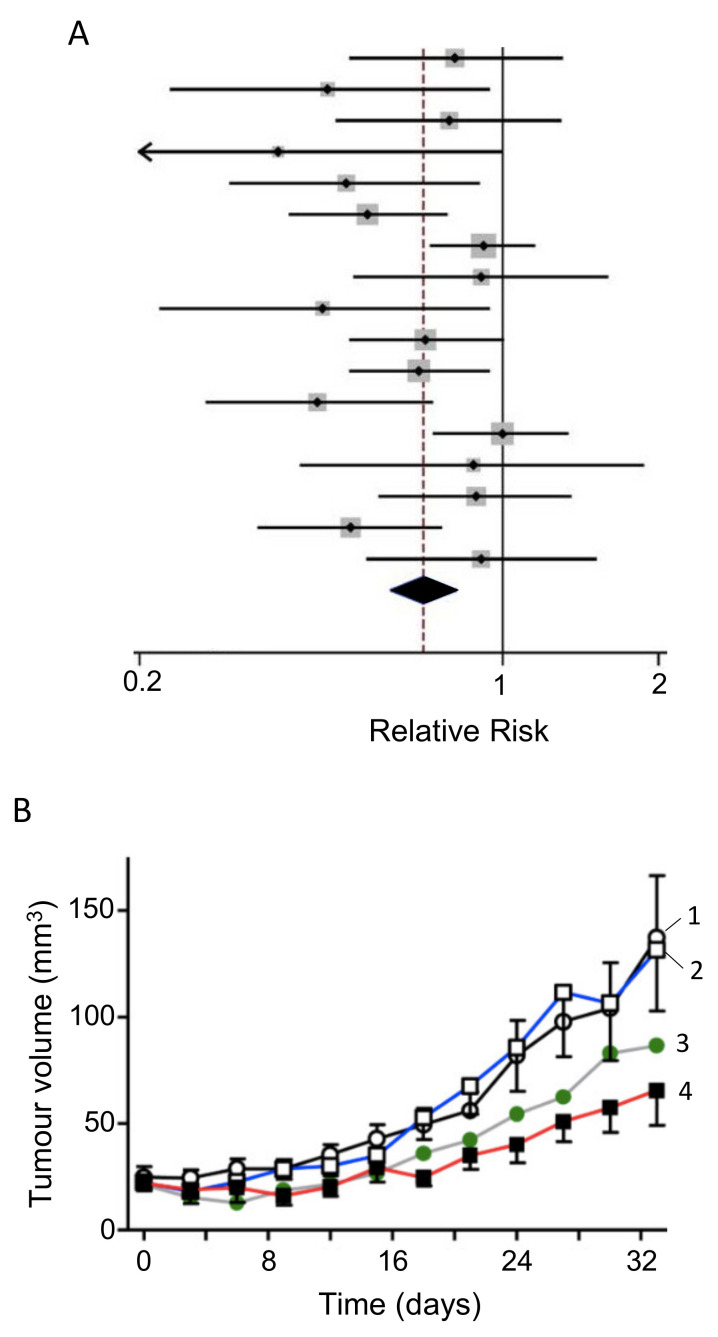
Effects of combining chemotherapy with a dietary compound: Vitamin C. (**A**) Forest plot comparing highest versus lowest categories of vitamin C intake and pancreatic cancer risk. Diamond indicates the average and the spread of the data and shows a beneficial effect of vitamin C intake. Modified from Fan et al. [200], where further details and primary data can be found. (**B**) Volume of Panc-1 xenograft tumors and the effects of gemcitabine treatment, with or without ascorbate. Data are expressed as the mean ± standard deviation (SD) of tumor volume. Curves indicate saline (1), gemcitabine (2), ascorbate (3), and gemcitabine + ascorbate (4). Combined treatment of gemcitabine and ascorbate decreased tumor volume by 52%, compared with only a 4% reduction in gemcitabine monotherapy. This difference was highly significant (*p* = 0.003). Modified from Espey et al. [203].

The essential characteristics of vitamin C (or ascorbic acid) and its potential usefulness against cancer, including PDAC, have recently been critically reviewed [195]. Humans cannot synthesize vitamin C, so it has to be consumed or taken as a supplement. Overall, it is ‘safe’ as it is water-soluble, and any excess can readily be extruded in urine. Vitamin C has two main modes of action that could ultimately lead to anti-cancer effects. First, it is primarily an antioxidant that may reduce the risk of developing cancer by preventing oxidative damage to normal cells [195]. Second, in the specific presence of catalytic metals, such as iron and copper, vitamin C may become a pro-oxidant and behave as an anti-cancer agent, e.g., via production of H_2_O_2_ [196,197]. Levels of both copper and iron tend to be higher in PDAC [198,199]. Accordingly, vitamin C may exert a protective role against PDAC. Interestingly, two meta-analyses reached different conclusions. Fan et al. [200] concluded from 17 observational studies that higher consumed levels of vitamin C intake were associated with a significantly reduced risk of PDAC (Figure 8A). On the other hand, Hua et al. [201] concluded that there was insufficient evidence to link vitamin C intake to PDAC risk. Importantly, such studies did not find any adverse effect of vitamin C on PDAC.

In early experiments on PDAC cells, treatment with ascorbate produced promising results [202]. In the in vitro experiments of Espey et al. [203], co-application with ascorbate (10 mM maximum) significantly increased the cytotoxicity of gemcitabine. In PDAC in vivo, also, intraperitoneal application of high-dose (4 g/kg body weight daily) ascorbate synergized with gemcitabine (at two different concentrations) and the tumor burden was halved (Figure 8B) [203]. Even in gemcitabine-resistant xenografts, ascorbate monotherapy produced a significant anticancer effect. In a further combination treatment, addition of ascorbate (catalyzed with manganese and applied subcutaneously or intraperitoneally) to gemcitabine enhanced the effectiveness of the chemotherapy on tumor growth and improved survival [196]. This study also showed that normal pancreatic ductal epithelial cells were not affected (in vitro) and there was no systemic oxidative stress in vivo [196]. In combination with radiotherapy, also, ascorbic acid demonstrated synergy. Thus, Alexander and Cullen [34] reported that the cytotoxic effect of radiation on PDAC cells in vitro can be potentiated by ascorbate (1 mM). The latter study also showed that ascorbate reduced the radiation-induced toxicity to normal tissues in vivo.

Importantly, two critical issues in ascorbic acid supplementation must be considered [195]. First, whilst plasma concentrations can reach a maximum of 100 μM when taken orally, it is i.v. administration that can result in concentrations of up to 100 mM [204,205]. It is these higher but still tolerable levels of ascorbic acid that will be beneficial against PDAC, especially the CSCs [205]. Similar synergistic effects of i.v. vitamin C and gemcitabine chemotherapy have also been seen in patients with triple-negative breast cancer, another difficult to treat cancer [206]. Second, vitamin C metabolism is different between mouse models and humans. In fact, humans need to obtain vitamin C from diet, whilst mice can synthesize it. Accordingly, care must be exercised in extending data from xenograft models to humans, and clinical trials would be essential. One phase I trial involving up to 100 g i.v. ascorbate in combination with radiotherapy was found to be safe (Table 1, C) [34]. Several other clinical trials aim to evaluate the effects of ascorbate, mostly in combination with chemotherapeutic agents (Table 1, D–F) [207,208]. Such studies could be facilitated by use of novel wearable biosensors of vitamin C (see Section 7.6).

In conclusion, vitamin C is usable in both a preventative setting and as an adjuvant in gemcitabine chemotherapy. Foods rich in vitamin C are listed in Table 2. Since this is a water-soluble vitamin, which the body can extrude any excess of, vitamin C supplements can be taken safely.

#### 3.3.3. Vitamin D

**Figure 9:**
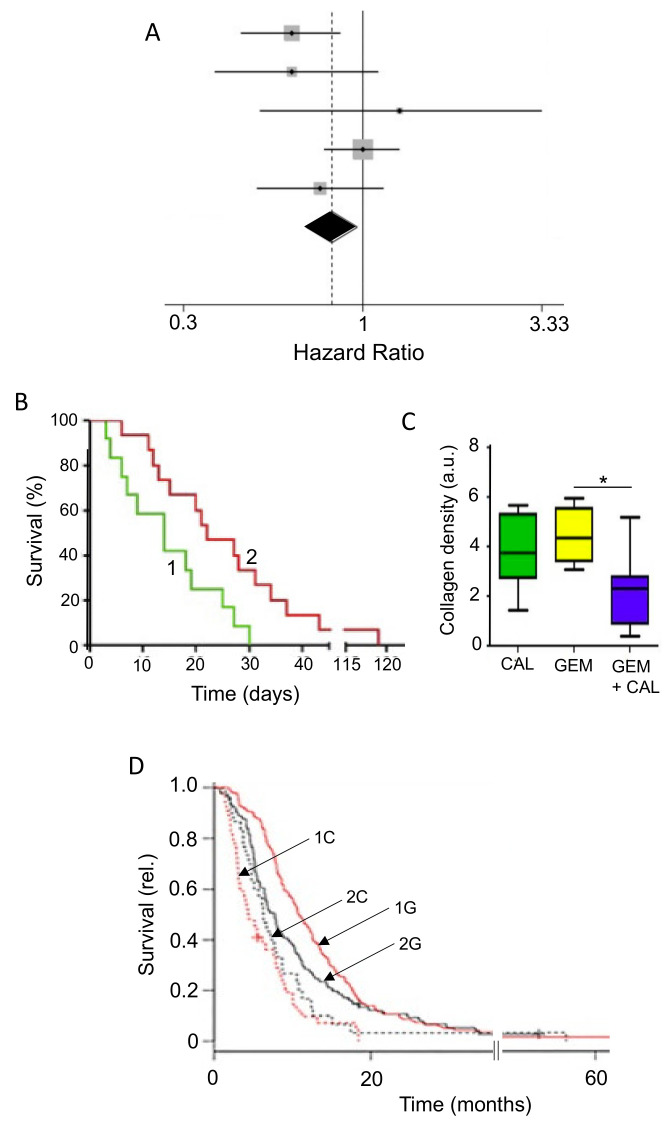
Effects of combining chemotherapy with a dietary compound: Vitamin D. (**A**) Meta-analysis of the association between plasma levels of 25-hydroxyvitamin D (25(OH)D) levels and pancreatic cancer mortality. Diamond indicates the average and the spread of the data, showing the beneficial effect of vitamin D. Modified from Zhang et al. [212], where further details and primary data can be found. (**B**) Kaplan–Meier survival analysis for KPC mice treated with gemcitabine (1) or gemcitabine + Calcipotriol (2). The effect of the combination was significantly greater than gemcitabine alone (*p* = 0.02) (*). Modified from Sherman et al. [218]. (**C**) Collagen density (arbitrary units). Effects of Calcipotriol (CAL), gemcitabine (GEM) and their combination (GEM + CAL), presented as non-parametric data. The combination was significantly more effective than gemcitabine alone (*p* < 0.05). Modified from Sherman et al. [218]. (**D**) Probability of survival of human pancreatic cancer patients with a mutated VDR/rs2853564 (curves indicated by 1), in comparison with wild type (curves indicated by 2). Each group was subject to treatment with gemcitabine (curves indicated by G). The effect of gemcitabine was significant only on the mutated VDR (*p* < 0.03 for 1 G v. 1 C), consistent with vitamin D improving the effectiveness of chemotherapy. Modified from Innocenti et al. [219].

Vitamin D concentrations in cancer patients are frequently found to be lower than in healthy individuals, with 75% of cancer patients exhibiting below normal levels [205,209]. Cohort studies on PDAC patients have shown both increased and decreased risk or no association with vitamin D levels [210,211]. On the other hand, a meta-analysis showed that higher vitamin D levels were significantly correlated with reduced risk of PDAC (Figure 9A) [212,213]. Combining chemotherapy with vitamin D has been under consideration for several years. Overall, the results of such combination treatments have been consistently beneficial [214,215,216,217]. This conclusion was verified and extended in an experiment on a mouse model, showing that supplementing gemcitabine with calcipotriol (a synthetic derivative of vitamin D) significantly increased median survival by some 50% (Figure 9B) [218]. 

Several modes of action have been associated with vitamin D. First, in relation to PDAC, binding of vitamin D to the vitamin D receptor (VDR) on PSCs inhibits their activation, thus leading to decreased desmoplasia and improved delivery of chemotherapy (Figure 9C) [56,218]. Second, induction of p21 and p27 cyclin-dependent kinase inhibitors resulted in reduced cancer cell proliferation [220]. Innocenti et al. [219] demonstrated a positive association between VDR expression and PDAC, with the overall survival of patients with the rs2853564 variant being more than doubled (Figure 9D). This variant of VDR would enhance the transcription of cell cycle arrest genes and thus suppress tumorigenesis.

In a recent study, using an in vivo orthotopic mouse model, Anbil et al. [221] demonstrated a novel “triple therapy” approach to PDAC. This involved combination of (i) the FDA-approved chemotherapeutic nanoliposomal irinotecan (nal-IRI) with (ii) activation of VDR using Calcipotriol, and (iii) ‘photodynamic priming’ (sub-threshold photodynamic therapy) within the fibroblasts of the tumor microenvironment (TME). This resulted in increased intra-tumoral accumulation of nal-IRI, (i) enabling some 75% reduction in the effective dose of nal-IRI, and (ii) suppressing tumorigenesis 5-fold more effectively (both effects significant).

Indirectly, by promoting the synthesis and binding of insulin (thereby decreasing insulin resistance and the risk of diabetes), vitamin D may also reduce the risk of PDAC [220,222]. Finally, a relationship between vitamin D deficiency and increased inflammation has also been suggested, but not confirmed [223]. It has even been highlighted that the daily recommended dietary allowance for vitamin D could be increased [224]. 

Interestingly, one study which demonstrated harmful effects of vitamin D linked it to concurrent low levels of vitamin A [211,225]. This emphasizes the importance of the body’s overall homeostasis and not just the levels of the individual biochemicals.

Several clinical trials evaluating the effects of vitamin D with chemotherapy (mainly gemcitabine) are currently underway (Table 1, G–K). An early randomized trial on patients with advanced pancreatic cancer receiving gemcitabine chemotherapy showed, rather surprisingly, that survival was not related to their vitamin D level [209]. This suggested that additional co-factors may need to be considered in such trials. A later, open-label phase I clinical trial, demonstrated that i.v. paricalcitol can be combined safely with gemcitabine in patients with advanced cancer [226]. Further trials involving i.v. treatments could produce significant benefit to patients, as demonstrated already for vitamin C (Section 3.3.2).

In conclusion, we consider vitamin D to be desirable against PDAC, both in the ‘background’ and during treatment. Vitamin D is obtained mainly from ultraviolet sunlight via synthesis in the skin (10–30 min of midday sunlight, several times per week, a little more for people with darker skin). Some foods that are relatively rich in vitamin D are listed in Table 2. Everyone should be aware of their vitamin D level and, if necessary, take a supplement.

#### 3.3.4. Vitamin E

**Figure 10:**
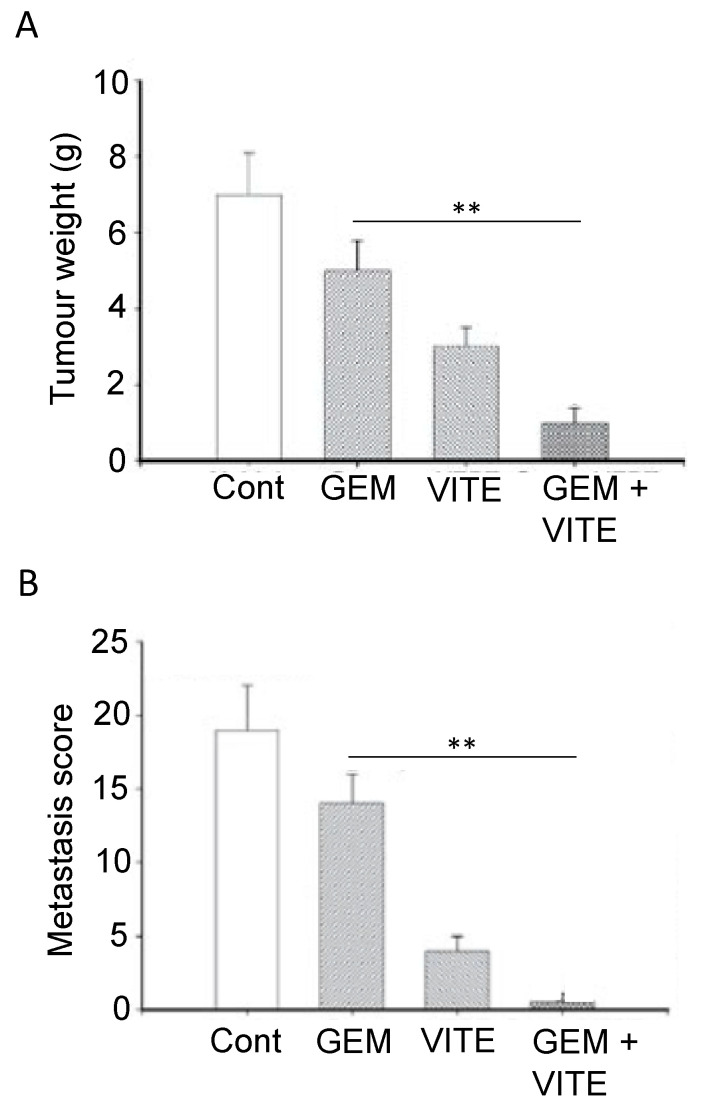
Effects of combining chemotherapy with a dietary compound: Vitamin E. (**A**) Tumor weights in an orthotopic xenograft mouse model of pancreatic cancer, treated with gemcitabine (GEM), delta-tocotrienol, a natural form of vitamin E (VITE) and their combination (GEM + VITE). Control (Cont) was olive oil. The effect of the combination was significantly greater than gemcitabine alone (*p* < 0.01) (**). (**B**) From the same experiment as in (**A**), showing the effect of the treatments on liver metastasis (scored as photons/cm^2^/second/steridian × 10^4^). The effect of the combination was significantly greater than gemcitabine alone (*p* < 0.01) (**). Modified from Husain et al. [230].

Vitamin E comprises four isomers, tocotrienols (TCTs) and, as an antioxidant, it could protect cellular DNA from free radical attack by increase the expression of the tumor suppressor p27 [85]. This could help inhibit the progression of PDAC. A meta-analysis of 10 studies covering the period 1991–2014 showed that consuming vitamin E would significantly reduce the risk of PDAC [227]. Gamma-TCT conjugated to gemcitabine produced an enhanced anti-PDAC effect (cell viability) in vitro [228]. In an in vivo orthotopic xenograft mouse of model of PDAC, combined application of gamma-TCT with gemcitabine reduced tumor volume more significantly (by greater than 50%) than gemcitabine alone [229]. This was substantiated in a later study (involving an orthotopic mouse xenograft model of stem-like PDAC cells), where delta-TCT in combination with gemcitabine reduced tumor weight significantly more than gemcitabine alone (Figure 10A) [230]. Also, liver (and lung) metastases were substantially suppressed (Figure 10B) [230]. In addition, the latter study revealed an anti-proliferative effect on the CSCs. Importantly, delta-TCT alone has also been subject to a phase I trial on PDAC patients (Table 1, L). This demonstrated that the supplement given to patients two weeks before surgery (i) was well tolerated and (ii) resulted in apoptotic cell death of the cancer cells [35]. 

In conclusion, the evidence for the anti-PDAC effects of vitamin E is so as to make useful during chemotherapy. Some dietary sources of vitamin E are listed in Table 2 [231]. If necessary, by medical advice, it can also be obtained as a supplement.

#### 3.3.5. Curcumin

**Figure 11:**
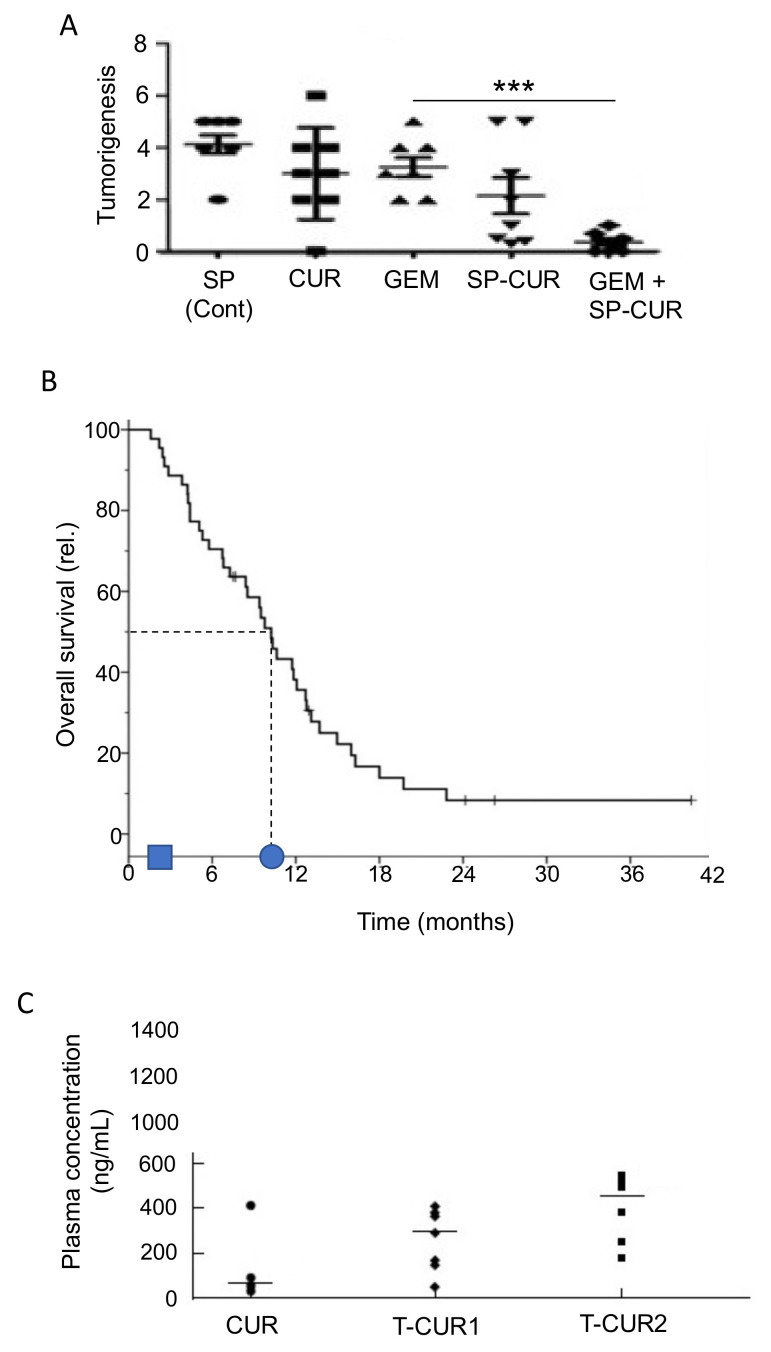
Effects of combining chemotherapy with a dietary compound: Curcumin. (**A**) An orthotopic mouse model of pancreatic cancer inoculated with fluorescent HPAF-II cells. The figure (dot plot) shows effects on tumor growth (deduced from fluorescence measurements) in mice treated with “superparamagnetic iron oxide nanoparticles” (SP, control), curcumin (CUR), gemcitabine (GEM), SP loaded with curcumin (SP-CUR) and a combination (SP-CUR + GEM). The effect of SP-CUR + GEM was significantly greater than gemcitabine alone (*p* < 0.0001) (***). Modified from Khan et al. [241]. (**B**) Overall survival determined within a group of 44 patients with locally advanced pancreatic cancer treated with a combination of gemcitabine and a curcumin combination (Meriva). The dotted lines indicate a median survival time of ca. 10 months (marked by the circle on the time axis). The average median survival time for such patients treated with gemcitabine alone would be ca. 2 months (marked by the square). Modified from Pastorelli et al. [242]. (**C**) Bioavailability of curcumin. Plasma curcumin levels following administration of conventional curcumin (CUR) and two doses of Theracurmin (T-CUR) were determined in patients. Points represent individual patients and horizontal bars correspond to median concentration values. 200 mg Theracurmin (T-CUR1) resulted in a median plasma concentration of 324 ng/mL, a 3.8-fold increase from the median 85 ng/mL produced by a much higher level of CUR (8 g). The bioavailability of Theracurmin was dose-dependent, 400 mg (T-CUR2) leading to 440 ng/mL. Modified from Kanai [232].

Curcumin, a chemical naturally found in turmeric, has been studied intensively and found to have considerable promise for prevention and treatment of PDAC, whilst having little effect on healthy cells [232,233,234]. In vitro studies have shown that curcumin re-sensitizes chemo-resistant PDAC cells and can inhibit growth of CSCs [235]. Several reports demonstrated the pro-apoptotic activities of curcumin [236,237]. Additionally, by inhibiting NF-κB signaling, curcumin was able to increase cellular uptake of gemcitabine and decrease desmoplasia, and thus decrease chemoresistance [238]. Preclinical trials have shown that curcumin can work both alone and in combination with chemotherapy to generate anti-PDAC effects [80,239]. In an early xenograft study, curcumin potentiated the effectiveness of gemcitabine in suppressing PDAC tumor formation significantly by ca. 67% [240]. In a more recent study, a nanoparticle form of curcumin was used and, again, was found to reduce tumor growth (as well as metastasis to several organs) and both effects were potentiated by gemcitabine co-application (Figure 11A) [241]. 

An initial clinical trial on PDAC patients who had developed gemcitabine resistance conducted by Kanai et al. [36] determined that curcumin alone could double the survival period from ca. 2.3 to 5 months (Table 1, M). These results were confirmed and extended by Pastorelli et al. [242], who showed that a novel combination of curcumin (Meriva) combined with gemcitabine increased median survival time further, on average from ca. 2 to 10 months (Figure 11B). Although well tolerated by patients, however, the effectiveness of curcumin could be limited by its low bioavailability. Prasad et al. [243] discussed several methods to increase the bioavailability of curcumin, the most promising one being the encapsulation of curcumin in hydrogel nanoparticles. To boost its clinical effectiveness, novel analogues have been developed with greater stability [244]. Compared with curcumin alone, theracurmin (a combination of curcumin and the gum ghatti polysaccharide) reached dose-dependently higher levels in blood, leading to significant improvement of fatigue in PDAC patients treated with gemcitabine (Figure 11C) [232,245]. More recently, Meriva, a formulation of curcumin and soy lethicin, resulted in fewer cases of neurotoxicity and hematoxicity in patients treated with gemcitabine [242].

In conclusion, curcumin has excellent properties against PDAC and could effectively synergize with chemotherapy [246]. Thus, patients should be generous with their intake of turmeric-containing meals, such as curries. Importantly, however, care must be exercised in ensuring that any curcumin supplement taken is ‘bioavailable’ and from a reliable source.

#### 3.3.6. Genistein

**Figure 12:**
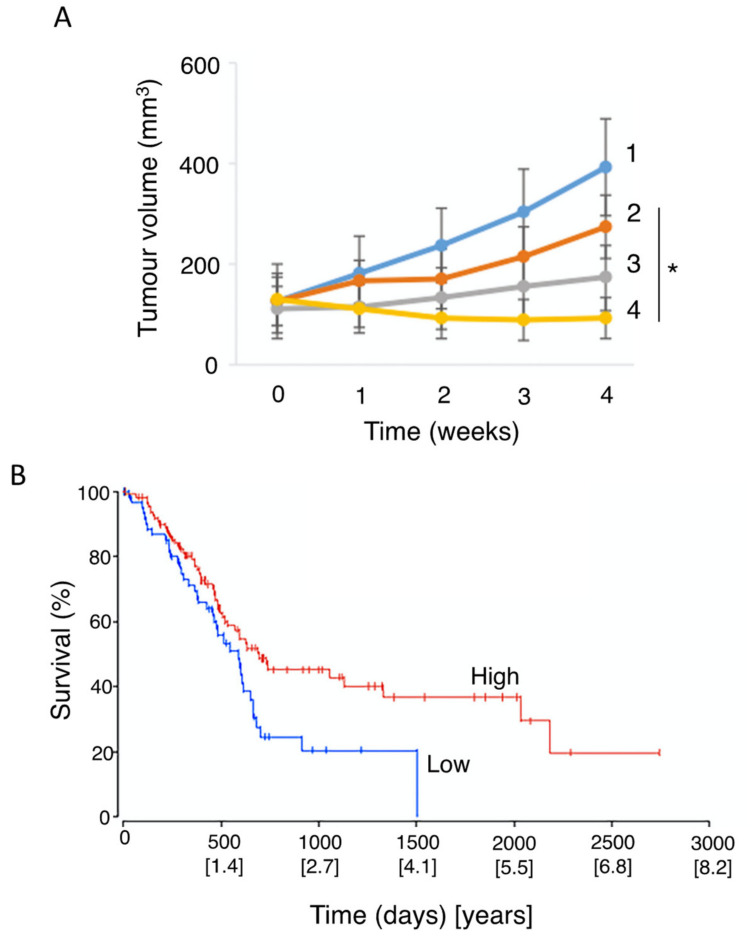
Effects of combining chemotherapy with a dietary compound: Genistein. (**A**) Pancreatic cancer from a patient-derived xenograft. Effects on tumor volume of treatment with gemcitabine (2), a genistein derivative, AXP107-11 (3), and their combination (4), compared with control (1). The combination was significantly more effective than gemcitabine alone (*p* < 0.05) (*). (**B**) Survival of pancreatic cancer patients in relation to the level of GPER1 mRNA, a possible molecular mechanism of genistein. Survival was expressed as a percentage of the total patient cohort (data from The Cancer Genome Atlas). Timescale is given in days and years. A relative high level of GPER1 mRNA expression was significantly associated with better overall survival (*p* = 0.019). Modified from Mesmar et al. [253].

Genistein is an isoflavone (and phytoestrogen, a class of plant-derived compounds with pro- or anti-estrogenic activity). It was reported to inhibit growth and invasiveness of PDAC cells by inducing cell cycle arrest and apoptosis [247,248,249]. These effects may partially have involved the CSC population [250]. Additionally, genistein potentiated the anti-tumor effect of 5-fluorouracil (5-FU), significantly, reducing tumorigenesis by inducing apoptotic and autophagic cell death in an in vivo mouse model of PDAC [251]. In gemcitabine-resistant PDAC cells in vivo, genistein (combined with an inhibitor of miR-223) suppressed invasiveness, by reversing epithelial-to-mesenchymal transition and prolonged survival [252]. Similarly, in a PDX model of PDAC, a genistein analogue, AXP107-11 (APX), showed gemcitabine-enhancing effects by reducing tumor growth (Figure 12A) [253]. Molecular analysis revealed the G protein-coupled estrogen receptor 1 (GPER1) to be one of the genes involved in the sensitizing effect of genistein on PDAC cells. Indeed, in a cohort of PDAC patients, a higher expression of GPER1 mRNA was associated with improved overall survival (Figure 12B) [253]. 

The compound AXP107-11 was adopted as a clinical candidate and proved to be safe in a phase I trial on chemotherapy-naïve PDAC patients, with some early signs of efficacy (Table 1, N) [37]. More broadly, synthetic isoflavones of genistein, triphendiol and phenoxodiol, have been proposed both as monotherapies and as sensitizers of chemotherapy (gemcitabine) for patients with early-stage and late-stage PDAC [254]. 

In conclusion, we consider genistein (and other isoflavones) to be appropriate for integration with gemcitabine chemotherapy. Soybeans and soy products are the richest sources of genistein and other isoflavones, such as apigenin, daidzin and glycitin (Table 2).

## 4. Nutraceuticals

Nutraceuticals are defined generally as natural substances that are not a part of normal diet but can be formulated and consumed as supplements. We have identified 3 nutraceutical compounds for which satisfy our criteria for therapeutic effects against PDAC and in some cases, the associated organs, especially the liver [255]. These are listed in Table 3 alongside their chemical formulae, main modes of action and natural sources.

### 4.1. Propolis

**Figure 13:**
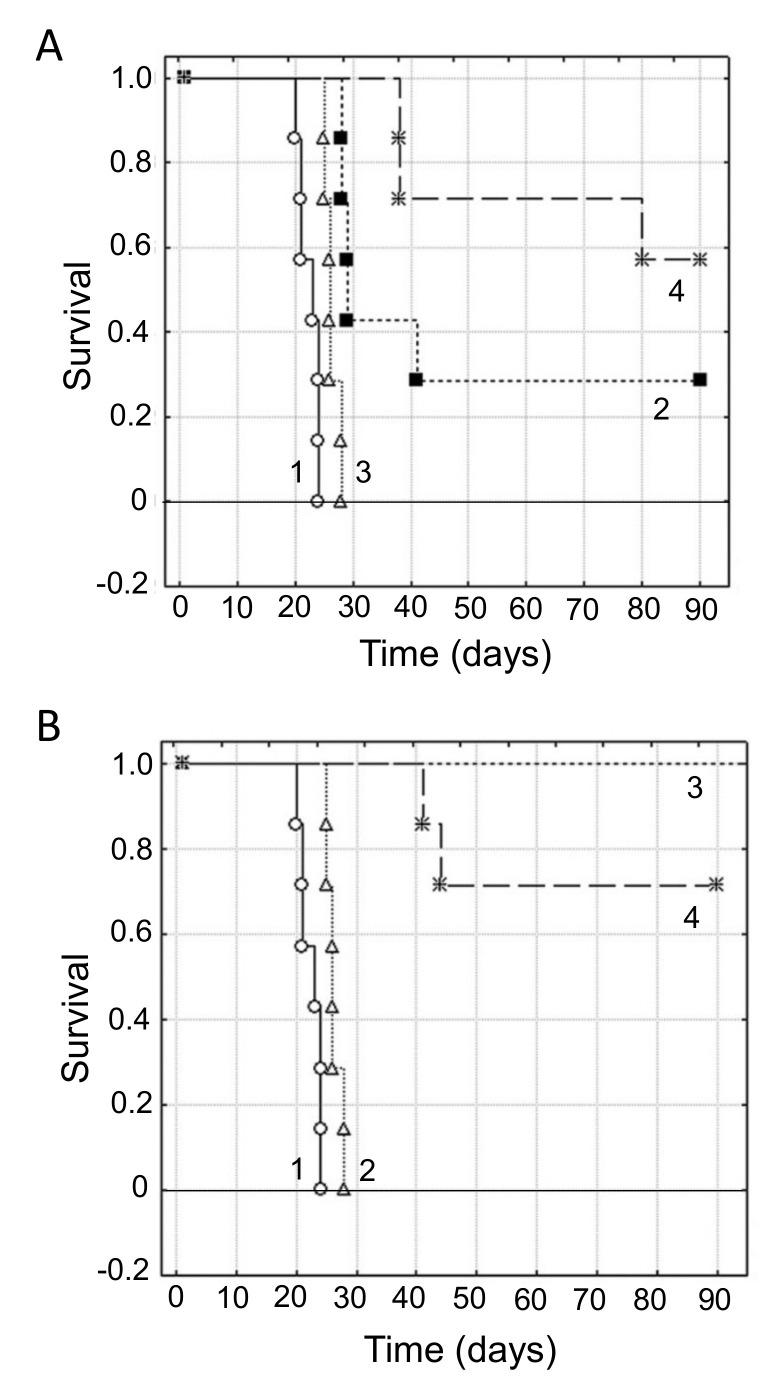
Effects of combining chemotherapy with a nutraceutical agent: Propolis. Kaplan–Meier analysis of survival of Swiss albino mice bearing Ehrlich ascites (mammary) tumors treated with a water-soluble derivative of propolis, WSDP (2), cisplatin (3) and their combination (4), compared with control (1). (**A**) 5 mg/kg cisplatin. There was a strong beneficial effect of the combination, compared with cisplatin treatment alone (4 vs. 3). (**B**) Treatment with 10 mg/kg cisplatin, which maintained complete survival over the duration of the experiment (3). Surprisingly, however, the combination produced an antagonistic effect, reducing the survival by some 25% (4 vs. 3). Modified from Oršolic et al. [260], where further details can be found.

This is a resinous mixture that bees make by combining sap of needle-leaved trees or evergreens with their own discharges and beeswax. Bees coat their hives with propolis for repair and protection against infections. Over 300 constituents of propolis have been identified to date, and propolis from different geographical locations and varying compositions exhibit a range of properties [256]. In particular, one of its active ingredients, caffeic acid phenethyl ester (CAPE), has been found to exert cytotoxic and anti-proliferative, even anti-invasive effects on various human cancer, including PDAC, cell lines and a mouse model [257,258,259]. Combination of a water-soluble derivative of propolis (WSDP) with cisplatin produced interesting effects on survival of tumor-bearing mice (Figure 13A,B) [260]. When combined with a relatively low dose of cisplatin (5 mg/kg), survival was approximately doubled by the combination with propolis (Figure 13A). This effect could additionally involve reduction of nephrotoxicity and hepatotoxicity produced by cisplatin [260]. In contrast, when combined with 10 mg/kg cisplatin, which by itself produced 100% survival, propolis produced an inhibitory effect (Figure 13B). Taken together, these results emphasize the importance of dosage in combination treatments. More recently, timing of the combination has also been highlighted as a contributing factor [261]. 

A small phase I study to determine the effectiveness of propolis in preventing radiotherapy-induced oral mucositis in head and neck cancer was completed in 2011, but the results are yet to be posted (Table 1, O)! Another phase II clinical trial evaluating the effectiveness of propolis on glycemic control in patients with type 2 diabetes mellitus without pharmacological treatment is ongoing (Table 1, P).

The evidence, taken together, including the beneficial effects on diabetes, kidney and liver, would suggest that propolis is an excellent anti-PDAC agent. Its natural source is the comb of honey, but it can most conveniently be taken as a supplement.

### 4.2. Triptolide

**Figure 14:**
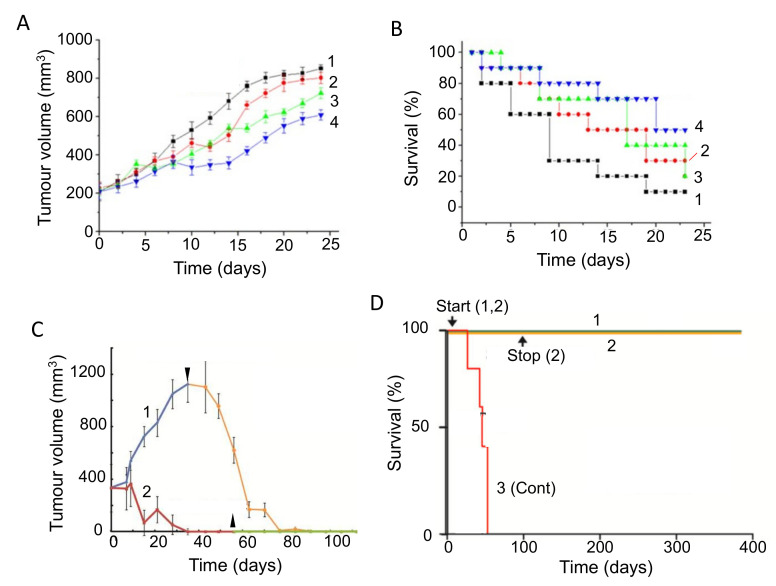
Effects of combining chemotherapy with a nutraceutical agent: Triptolide. (**A**) Time course of tumorigenesis in xenografts of pancreatic cancer, treated with gemcitabine (2), and two different triptolide analogues, TP-PM (3), AS-PPT (4). Control data are shown in (1). Compared with gemcitabine, the effects of TP-PM and AS-PPT were significant (*p* < 0.05 and *p* < 0.01, respectively). (**B**) Survival rate data from the experiments in A. Compared with gemcitabine, the effect of AS-PPT was highly significant (*p* < 0.01). (**A**,**B**) modified from Wang et al. [267]. (**C**). Time course of tumorigenesis (tumor volume) in subcutaneously implanted patient-derived xenografts of pancreatic cancer, treated with minnelide. Application of minnelide (downward-pointing arrow) reversed the tumorigenesis (curve 1). In the other experiment (curve 2), application of minnelide at the same time as the tumor induction prevented tumorigenesis, and this was maintained even after minnelide was removed (upward-pointing arrow). (**D**) Survival data (expressed as a percentage of total) determined in an orthotopic xenograft mouse model of pancreatic cancer. Survival was 100% in animals with both maintained application of minnelide (1) and removal some 100 days afterwards (2). In contrast, under untreated control conditions (3, Cont), survival was reduced to 0% by day 50. In experiments 1 and 2, minnelide application started at the same time as the induction of the tumor. (**C**,**D**) modified from Banerjee and Saluja [271].

This is a diterpenoid, the main active ingredient isolated from the herbal plant ‘thunder god vine’ (*Tripterygium wilfordii*). It has been used for centuries in traditional Chinese medicine to treat immune-related disorders. In addition to its anti-inflammatory and immunosuppressive activities, triptolide possesses potent anti-tumor properties [262]. Several studies have shown that triptolide is effective both in vitro and in vivo against PDAC cells (including CSCs and those derived from patients) by inducing apoptosis and autophagy [262,263]. Triptolide has also been shown to enhance the effect of TRAIL (tumor necrosis factor-related apoptosis-inducing ligand) by downregulating PUM1, an RNA-binding protein involved in translational regulation [264]. There is also promising evidence, in vitro and in vivo, that triptolide sensitizes the effect of gemcitabine on PDAC cells, including those that are drug-resistant [265,266]. The effectiveness of the combination treatment was associated with the *KRAS* status of PDAC cells derived from patients, with cell viability being suppressed significantly more if mutation was present [262]. Importantly, in a xenograft model of PDAC, a nanoparticle formulation of triptolide with improved tissue accumulation enhanced the effect of gemcitabine on tumor suppression and survival (Figure 14A,B) [267]. In another nanoparticle formulation, significantly increased effectiveness of triptolide has also been achieved by loading into silk fibroin [268].

The potential use of triptolide as a clinical agent against PDAC (and liver cancer) has been highlighted by the development of a ‘pro-drug’, minnelide [262,269]. In vivo, minnelide decreased tumorigenesis in a highly metastatic orthotopically-induced human PDAC model [270]. In another in vivo study, application of minnelide reversed tumorigenesis and suppressed it from the beginning if co-initiated (Figure 14C) [271]. Furthermore, the effect of minnelide persisted even after treatment was stopped. Consistent with these effects, minnelide (even when applied transiently) led to 100% survival in tumor-bearing mice (Figure 14D) [271].

Minnelide is currently being evaluated in a phase II clinical trial against gemcitabine-refractory PDAC (Table 1, Q) and a phase I clinical trial aiming to determine its safety in combination with nab-paclitaxel (Table 1, R).

The evidence, taken together, would strongly suggest that triptolide can be incorporated effectively into integrated management of PDAC. The natural source of triptolide is thunder god vine (Table 3). However, it would most safely be taken as a supplement.

### 4.3. Cannabidiol

**Figure 15:**
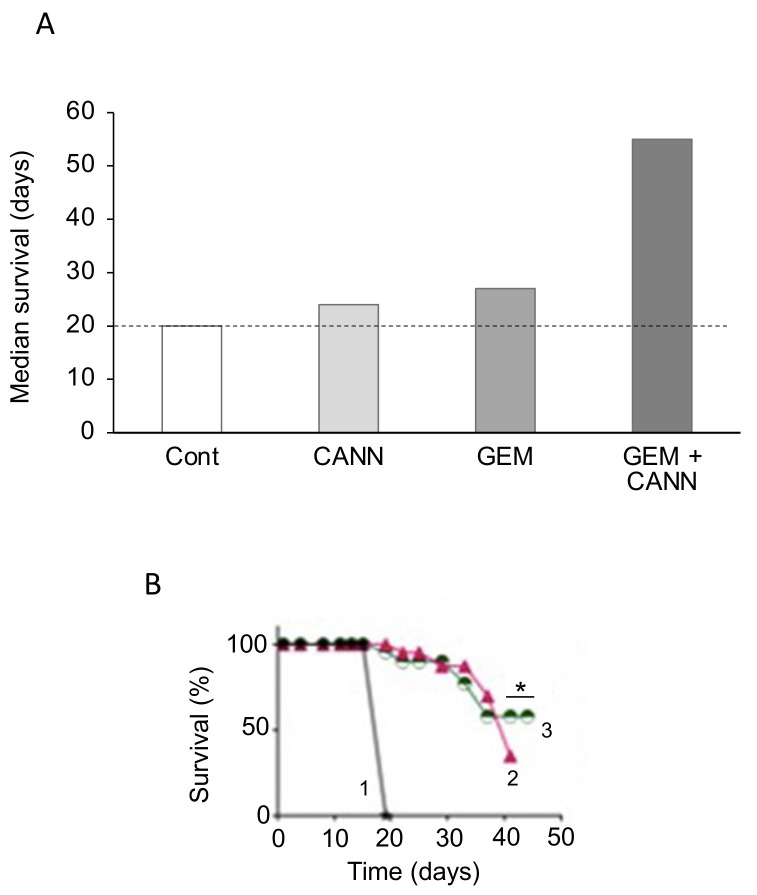
Effects of combining chemotherapy with a nutraceutical agent: Cannabidiol. (**A**) Survival data (median values) for KPC mice treated with cannabidiol (CANN), gemcitabine (GEM) and their combination, in comparison with control (Cont) marked by the dotted horizontal line. Compared with Cont, the combination produced a significantly bigger effect. Although there was a noticeable difference between the effects of the combination and GEM, statistical analysis was not available. Data obtained from the Kaplan–Meier analyses of Ferro et al. [277], where further details can be found. (**B**) In vivo treatment of mice (KPC model) with a non-psychoactive derivative of cannabis, FBL-03G (2), combined with radiation therapy (3), compared with control (1). A radiosensitizer was present in the background. A significantly beneficial effect on survival of the combination was observed after some 40 days of treatment (*p* < 0.05, 3 vs. 2) (*). Modified from Moreau et al. [278].

Medical use of cannabis was introduced to Western medicine in the 19th century. Currently, cannabis is listed as a schedule I controlled substance, meaning that there is high potential for abuse and a lack of evidence with regards to medical safety [272]. As a result, the evaluation of potential medical, including anti-cancer, benefit of cannabis has been under intense discussion [273,274]. Recent experiments have demonstrated that compounds isolated from cannabis (cannabinoids) can have anti-cancer effects, alongside their pain-relieving activity. As such, cannabidiol (CBD) and tetrahydrocannabinol (THC) are nutraceuticals of significant current and growing interest. THC is the primary psychoactive compound found in cannabis, while these effects of CBD are limited. Both compounds are able to inhibit PDAC cell activity in vitro by promoting apoptosis and inhibiting proliferation [275]. Early in vivo studies on xenograft models of PDAC showed synergism between cannabinoids and gemcitabine, associated with increased autophagy [276]. In PDAC xenografts, combined treatment of mice with CBD and gemcitabine decreased cell proliferation and doubled median survival (from ca. 27 to 55 days), compared to gemcitabine monotherapy (Figure 15A) [277]. The effect of CBD occurred via inhibition of the G protein-coupled receptor GPR55 and MAPK signaling. A flavonoid derivative of cannabis, FBL-03G (mixed with a radiosensitizer) enhanced the effect of radiotherapy on PDAC in vivo, significantly delaying metastasis and improving survival (Figure 15B) [278]. The effect of a mixture of THC and CBD on cachexia is being evaluated in a phase II clinical trial (Table 1, S). Additionally, dronabil, a synthetic form of THC, has been approved by the FDA for treatment of nausea and vomiting in cancer patients that are non-responsive to chemotherapy [https://www.accessdata.fda.gov/drugsatfda_docs/nda/2016/205525Orig1s000Approv.pdf]. A phase III clinical trial aims to determine the effect of dronabinol on FOLFIRINOX and gemcitabine-related side effects in metastatic PDAC patients (Table 1, T).

The evidence, taken together, would strongly suggest that cannabidiol has excellent anti-PDAC properties, in relation to both potentiating treatments and reducing side effects. Its natural source is the cannabis plant (Table 3). However, it should be taken as a supplement.

## 5. Lifestyle Factors

There are several non-genetic factors that can affect the risk of developing PDAC. Men are 30% more likely to develop the disease than women, possibly due to hormonal effects. Diabetes and obesity are also major contributing risks. Lifestyle and environmental factors include smoking, high alcohol consumption, lack of exercise and a poor diet [79,225,279]. Some 10% of PDAC cases are associated with a family history, ~12% are associated with obesity and ~29% are estimated to be caused by smoking [280]. As a cancer of affluence, developed countries have higher age-standardized incidence of PDAC, which may be attributable to an increased exposure to these risk factors [281]. Industries associated with chlorinated hydrocarbons, such as dry-cleaning and metal-related work, have been suggested as occupational hazards for PDAC [282]. As regards molecular factors, PDAC generally is characterized by a number of germline or genetic mutations, with the most common genes involved being *KRAS* (90%), *CDK2NA* (90%), *TP53* (75–90%) and *DPC4/SMAD4* (50%) [283]. Whole-genome studies recently associated a truncating mutation in the oncogene *RABL3* with PDAC development [284]. Studies in the USA have shown that black Americans are the racial group at highest risk, with a mixture of genetic and modifiable risk factors. Interestingly, those with type A, AB or B blood groups are at a higher risk, although the link between blood type and PDAC has yet to be fully explained [279]. Overall, a large-scale study concluded that adherence to a healthy lifestyle was inversely associated with PDAC risk, even when smoking, a major risk factor, was not considered [285].

### 5.1. Obesity

Obesity is classified as having a body mass index (BMI, ratio of weight in kilograms to height in meters squared) score of over 30. The prevalence of obesity worldwide has increased steeply in recent decades and presents serious health problems [286,287,288]. Obesity has also been significantly associated with increased risk of PDAC [289,290,291]. Indeed, meta-analyses showed that BMI and PDAC risk are positively correlated [292]. The correlation is ‘dose-dependent’, with statistically significant trends of increasing risk of PDAC with increasing BMI, and this was observed in all models [292]. Obesity would lead to increased adipose tissue in and around the pancreas, increasing the desmoplasia and causing an inflammatory and drug-resistant microenvironment [292,293,294]. Such chronic inflammation would be immunosuppressive and has been suggested to promote PDAC [295]. 

The impact of obesity on PDAC development and progression has been studied and confirmed in vivo [294]. Thus, it was shown in syngeneic mouse models of PDAC that primary tumors grew to significantly larger sizes in both diet-induced and genetically induced obese mice compared with lean mice. In addition, numbers of mesenteric peritoneal and retroperitoneal metastases were significantly higher in obese mice. Similarly, a high-fat diet has been associated with increased PDAC risk [293]. In terms of intensive weight management, a recent study showed that low-carbohydrate diets had similar outcomes as with medication [296]. In terms of chemotherapy (5-FU), PDAC patients with BMI ≤ 25 were found to have a significant survival benefit (median survival increasing significantly by some 50%) which was not seen in patients with BMI > 25 [294]. 

We should also note that low microbiome diversity has long been associated with an increased risk of obesity [293]. Importantly, in parallel, it was recently found that high tumor microbiome diversity in PDAC was associated with long-term survival [297]. By modulating the tumor immune response, a diverse microbiome can reduce PDAC progression and improve survival of patients. A diet that promotes the thriving of a multitude of gut bacteria may therefore benefit PDAC patients [297].

Finally, we should highlight the fact that obesity and diabetes (Section 5.2) are closely associated. Indeed, secretion of adiponectin, leptin and other factors by adipose tissue can induce insulin resistance, thereby promoting diabetes [298]. Furthermore, a recent cohort study on a large Danish population concluded that the impact of obesity on diabetes risk is more important than genetics [299]. 

### 5.2. Diabetes

**Figure 16:**
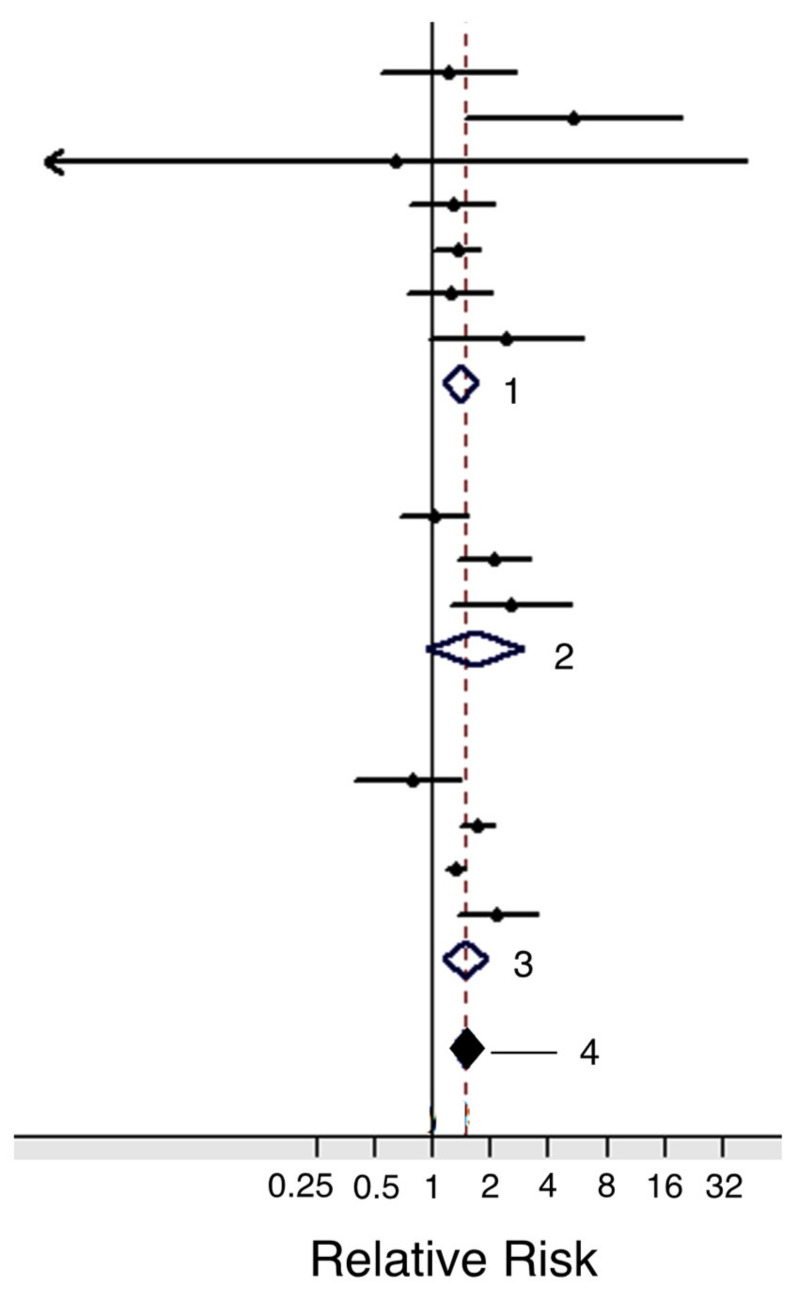
Assessment of diabetes as a risk factor for pancreatic cancer. Forest plot showing positive association between diabetes (lasting ≥ 10 years) and the risk of pancreatic cancer. Data sets from three different studies are presented: case-control (1), nested case-control (2) and cohort (3) studies; individual data averages are indicated by diamonds. The overall average is indicated by the black diamond (4). The associations were found to be significant in all the analyses. Modified from Song et al. [300], where further details and primary data can be found.

The impact of diabetes on PDAC has already been discussed in part in relation to GI (Section 3.1.2). Type II diabetes is well known to co-occur with PDAC and, indeed, an extensive meta-analysis has confirmed that long-term diabetes (>10 years) has a significant association with increased PDAC risk (Figure 16) [300]. Also, about 74% of PDAC patients are diagnosed with diabetes two years prior to their diagnosis [295]. About 25% of patients diagnosed with PDAC have diabetes at the time of diagnosis [301]. Links have also been shown between diabetes and jaundice, which is one of the most visible symptoms of PDAC representing liver damage [302].

An association between high blood glucose and PDAC incidence, particularly in women, has been suggested [85]. Increased cancer risk has been seen in patients with elevated levels of glucose even below what is considered to be the diabetic threshold. It has also been suggested that every 0.56 mmol/L (ca. 10%) increase in the blood glucose level (the average level is around 5 mmol/L) could cause some 14% increase in PDAC risk [303]. This positive relationship has been attributed to the fact that PDAC cells have a higher requirement for glucose than their healthy equivalents, and so the hyperglycemia resulting from type II diabetes provides more nutrients to enable their growth [303]. Additionally, such hyperglycemia may encourage development of stem cell characteristics [304]. Interestingly, high-GI diets that elicit high levels of insulin may also reduce the effectiveness of anti-cancer drugs by activating signals downstream to PI3K [305]. This highlights further the damaging effect of diabetes on PDAC and would advocate for the adoption of a low-GI diet, reducing the levels of glucose in the body [306]. 

Interestingly, the relationship between diabetes and PDAC goes both ways, whereby diabetes is associated with an increased risk of PDAC. Indeed, diabetes is often improved following surgical resection, if this is a viable form of treatment for the PDAC patient in question [298]. 

Recently, alternate day fasting was shown to reduced insulin resistance in patients at high risk of developing diabetes [307]. Although, daily calorie restriction also reduced the weight of participants, the same benefits on insulin resistance were not observed. Work on in vivo murine and human models of PDAC has shown that a low-calorie diet can decrease expression of inflammation-related genes and tumor growth [308]. It has also been suggested that calorie restriction reduces the development of pre-PDAC lesions [288]. Finally, “fasting mimicking diet” was recently shown to improve the effectiveness of chemotherapy [309]. 

### 5.3. Smoking

Tobacco products (cigarettes, cigars, pipes) contain many known carcinogens and some of these have been linked directly to PDAC [310,311,312]. Some risks may be mediated indirectly through diabetes and pancreatitis [313,314]. Overall, the evidence is consistent in showing increased risk of PDAC from smoking, with risk being highest for ‘current’ smokers, less for ‘former’ smokers and least for ‘never’ smokers [315]. At present, there is no reliable information as regards e-cigarettes for PDAC specifically, but evidence from other cancers would suggest caution, especially since nicotine could be present [316]. 

Several meta-analyses showed that tobacco usage significantly increases PDAC risk, by as much as two-fold [310,311,312,315,317]. The risk increased with the number of cigarettes smoked and duration of smoking, although the relationship may not be linear [310,312]. Heavy smokers were most at risk, with those smoking more than 30 cigarettes per day having the highest risk for developing PDAC. They were also likely to inhale the smoke more deeply and so suffer a greater impact from the carcinogen, nicotine-derived nitrosamine ketones. More than 20% of PDAC cases are the direct result of induced carcinogenesis or the indirect consequence of developing chronic pancreatitis. Smoking may also lead to an earlier onset of PDAC for those already predisposed to the disease. Since the tobacco smoke itself is damaging, passive smokers are also at risk [318]. 

Several mechanisms have been associated with the potentiating effect of smoking on PDAC. Cigarette smoke exposure impairs β-cell functioning through activation of oxidative stress, suppressing β-cell proliferation and impairing normal insulin production, processing and secretion [319]. Another study showed nicotine to enhance the proliferation, migration and invasion of human PDAC cells via activation of an atypical protein kinase C [320]. Importantly, also, nicotine and cigarette smoke extract were found to increase the stemness of PDAC, through transcriptional signaling, and thus accelerate tumor growth [321,322]. 

Patients are therefore advised to completely stop smoking once diagnosed with PDAC. Some studies suggested that smokers would carry the risk even after giving it up for 15 years, with the risk of PDAC reaching the level of never smokers only after some 20 years [310,323]. 

### 5.4. Alcohol

Excessive alcohol consumption can lead to a variety of health complications, including increased risk of PDAC. Indeed, several studies have shown repeatedly that alcohol consumption and PDAC are significantly positively correlated [323,324,325,326,327,328,329]. One study found no significant association between PDAC risk and either overall alcohol consumption or type of alcohol consumed [330]. Possible reasons given for this apparent inconsistency included the relatively small size of the sample and the uncertainty of the control ‘non-drinkers’ group. Independently, it has also been established that about one-half of the cases of PDAC are due to the impact of alcohol on pancreatitis [331]. Furthermore, alcohol consumption has detrimental effects on the physiology of the liver, which is closely associated with PDAC [332]. 

Acetaldehyde, ROS and other toxic metabolites generated by the oxidation of ethanol may affect the endocrine and exocrine functions of the pancreas. These metabolites may cause oxidative stress, cell damage and increased activity of P450 enzymes, thus activating PSCs and releasing pro-inflammatory mediators causing fibrosis [333]. Consequently, these effects can lead directly to carcinogenesis or can result in chronic pancreatitis [279]. Alcohol may also impact on CSCs due to acetaldehyde- and/or ROS-induced DNA damage, thus promoting their self-renewal within a (de)regulated microenvironment [334]. 

**Figure 17:**
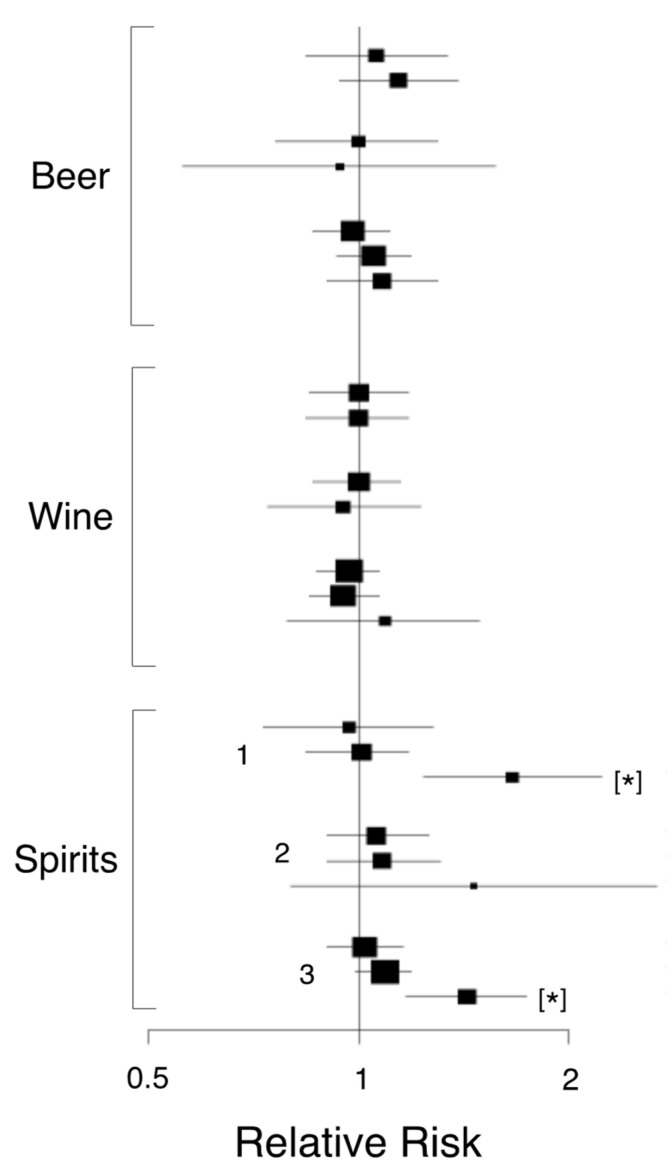
Effects of alcohol consumption on pancreatic cancer risk. Risk of pancreatic cancer associated with intake of different types of alcohol (beer, wine and spirits). The relative risks were significant for spirits in men (1) and combination of men and women (3), but not women alone (2). Significance is indicated by asterisks (*p*-values not specified). Modified from Wang et al. [335], where further details and primary data can be found.

The increased risk of PDAC due to alcohol intake depends on the type, strength and duration of consumption as well as gender [326,335]. A meta-analysis of 19 studies concluded that high alcohol use (greater than 3 units per day) increased the chance of developing PDAC, whereas low to moderate levels had no effect [335]. Interestingly, no risk seemed to be associated with wine or beer, probably due to their lower alcohol content and/or presence of potentially beneficial micronutrients (Figure 17). The effect of alcohol was more pronounced in men, thereby also highlighting the possible importance of hormonal and/or metabolic factors.

In conclusion, strong alcoholic drinks should be avoided by PDAC patients but limited red wine (containing resveratrol) and light beers may be enjoyed without overt damage.

### 5.5. Exercise

Substantial evidence increasingly suggests that physical activity is significantly beneficial to cancer, including PDAC, patients. Pre-clinical evidence suggests that exercise is useful as an adjuvant to chemotherapy by improving drug delivery and antitumor efficacy, in part by remodeling tumor vasculature [336]. This would be especially important for PDAC due to the presence of the dense stromal component (“hyperplasia”) that compresses vasculature, stunts blood vessel growth and reduces circulation, preventing the effective delivery of drugs to the tumor cells. Therefore, one possible method for improving outcomes for patients receiving standard chemotherapy is to improve the vasculature within the tumor. In addition, exercise can reduce fatigue, fitness loss, as well as improve mental health and the psychosocial downside of treatment [38]. 

**Figure 18:**
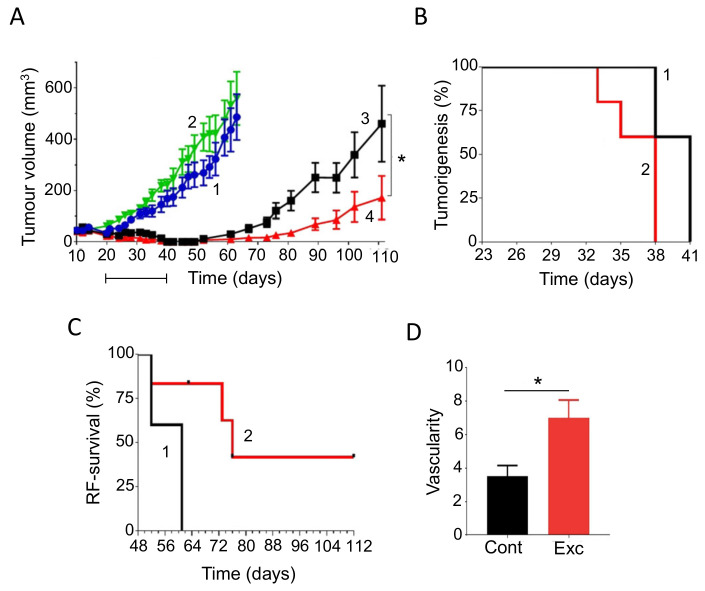
Effects of combining exercise with chemotherapy. (**A**) Exercise significantly improved gemcitabine efficacy in suppressing regrowth of tumors (measured as “tumor volume”). Graphs denote the following conditions: 1, control; 2, exercise alone; 3, gemcitabine; 4, gemcitabine with exercise. The application period of gemcitabine is indicated by the short horizontal bar. Data are presented as means ± standard error. Whilst exercise alone did not produce an effect, it did potentiate the suppressive effect of gemcitabine (*p* = 0.03 for 4 vs. 3). (**B**) Time course of tumorigenesis (percentage of tumor-bearing mice). Time is given as days when a tumor could be measured. Mice treated with gemcitabine plus exercise (2) achieved tumor-free state significantly earlier (*p* = 0.02), compared with mice treated with gemcitabine alone (1). (**C**) Recurrence-free (RF) survival of mice. Treatment with gemcitabine alone (1) was associated with significantly shorter survival (*p* = 0.01), compared with mice treated with gemcitabine plus exercise (2). (**D**) Effects of coupling exercise (Exc) with treatment (chemo/radiotherapy) on vascular remodeling. Vascularity was quantified as the number of vessels within ×200 fields of view. Tissues from patients who underwent an exercise (“prehab”) period were compared with control (Cont) data from archival material. Histobars show means ± standard error (*n* = 15 and 23, for historical controls and prehab patients, respectively). The effect of exercising was highly significant (*p* = 0.01) (*). Modified from Bedoya et al. [38].

In one detailed study on a mouse xenograft model of PDAC, the combination of gemcitabine plus treadmill walking inhibited tumor growth significantly more than gemcitabine alone [336]. The same effect was seen in a PDX model of PDAC (Figure 18A) [38]. The latter study also showed that the time taken for tumors to regress was significantly (ca. 10%) shorter for the mice treated with the combination compared with gemcitabine monotherapy (Figure 18B). Finally, the recurrence-free survival was extended significantly by some 21% by the combination treatment compared with gemcitabine alone (Figure 18C). This greater inhibition depended on vascular remodeling after exercise, which correlated with more chemotherapy delivery to tumors [336]. When vascular remodeling was prevented pharmacologically or genetically, the beneficial effect of exercising was lost.

The benefits of combining treatment with exercise were confirmed in a pilot study on PDAC patients (Table 1, U). Thus, 70 patients with potentially resectable PDAC were prescribed an exercise regime concurrently with preoperative chemotherapy and/or chemoradiation [38]. Of the 70 patients enrolled, 33 (47%) underwent resection of their primary tumor. Tumor tissue and activity data could be collected for 23 of those 33 patients and these comprised the “prehab” group. These patients’ tumor samples were compared with 13 historical “control” tumors obtained from patients who underwent surgery following chemotherapy or chemoradiation therapy and who had not participated in a prescribed exercise program. In the two groups, the patient demographics, disease stage, treatment and time from pathological diagnosis to surgery were not significantly different. Also, there was no differences in tumor stage, lymph node status, tumor regression grade, stromal and cellular components, as well as the degree of fibrosis or inflammation. Importantly, the results showed that the tumor vasculature from prehab patients underwent remodeling, becoming twice as vascularized, compared with controls (Figure 18D). Furthermore, these vessels were significantly more elongated and had wider lumens.

In conclusion, animal models and human studies agree in showing that exercise can enhance the effectiveness of clinical treatment of PDAC. As regards, how much exercise would be optimal, in the study of Bedoya et al. [38], this involved at least 120 min of moderate-intensity, home-based exercise (60 min of aerobic exercise and 60 min of ‘strengthening’ per week). The “prehab” group had exercised for a mean of ca. 15 weeks and reported an average of ca. 170 min per week of aerobic and strengthening exercise prior to surgery. The National Comprehensive Cancer Network (an alliance of 30 leading cancer centers) recommends starting slowly and progressing incrementally. Patients can also choose the kind of exercise that would suit them the best. Most recently, the American Cancer Society recommended 2.5 to 5 hours of moderate-intensity or 1.25 to 2.5 hours of vigorous-intensity exercise per week [337]. Depending on fitness and comfort level, some people may want to start simply with a 10 min walk around the block, others may find they can exercise for 20 min (or longer) right away [https://www.pancan.org/news/exercise–tips–people–pancreatic–cancer/]. In any case, exercising would best be done, if possible, in green spaces [338]. 

## 6. A Scheme for Integration of Clinical and Complementary Approaches: Treatment Logistics and Strategy

In this review, we have identified as having anti-PDAC characteristics (i) a range of background dietary conditioners and lifestyle factors, (ii) 6 specific dietary compounds which can also be taken as supplements and (iii) 3 nutraceutical compounds. These were chosen according to evidence-based criteria ranging from clinical trials to in vitro experiments. Based on the assembled evidence, a number of recommendations may now be made for possible integration of these ‘complementary’ measures into conventional treatments with a view, ultimately, to improving patient outcome. We have focused on gemcitabine chemotherapy as first-line treatment. 

First, we should stress that, as with any sickness, prevention is best [339,340]. All the remedies that we have highlighted here can be taken by individuals with any risk of developing PDAC. High-risk individuals would include those who are genetically predisposed and/or have certain professions and lifestyles. The same would be true for patients following their treatment, including excisional surgery for PDAC, i.e., taking the measures discussed could also work against possible recurrence.

The crucial period is during chemotherapy. Whilst the proposed integration will be aimed primarily at improving the effectiveness of the treatment, it is also important to consider that undesirable side effects, at least in some cases, may also be reduced. In deciding the way forward, we admit that not every combination has been tested on patients and much more research is needed before fully integrated management of PDAC becomes a routine clinical reality. Nevertheless, any benefit gained by the patient at this stage would be welcome. What follows are recommendations for how best integrated management of PDAC may be achieved. We consider the integration as comprising ‘general conditioning’ and ‘specific additions’ to the main gemcitabine chemotherapy.

### 6.1. General Conditioning

This involves a number of ‘background’ measures that can be adopted to help reduce the risk of PDAC developing, progressing or recurring. Overall, these measures relate to diet and lifestyle. We recommend organic diets that are low-GI, low-cholesterol and high-alkaline, avoiding especially red meat and dairy. Many of the dietary approaches complement each other. For example, eating more fruit and vegetables whilst also cutting back on red meat, would increase the body’s alkalinity and reduce the level of cholesterol. It may also lessen the chances of ingesting carcinogens from what may be non-organic meat itself.

Lifestyle should include limited alcohol intake, no smoking and regular exercise and avoiding obesity. In addition, patients could derive significant benefit, as regards both the cancer and its side effects, from activities like yoga, meditation/mindfulness, pilates, tai chi, acupuncture, music therapy and art therapy [341]. These latter topics are beyond the scope of the current review.

Adopting these integrative measures promises to improve ongoing treatment, as well as reduce (i) the risk of PDAC directly and (ii) many of the associated risk factors (including diabetes, inflammation and pancreatitis) indirectly. Such measures can also be beneficial to patients who may initially be denied surgery (e.g., due to their general state of health or obesity) but may eventually become eligible.

In the following, we give recommendations for how best to integrate specific dietary and nutraceutical agents with gemcitabine chemotherapy. Further work is required to elucidate whether the kinds of complementary measures considered would generate synergy also with FOLFIRINOX chemotherapy.

### 6.2. Specific Additions

**Figure 19:**
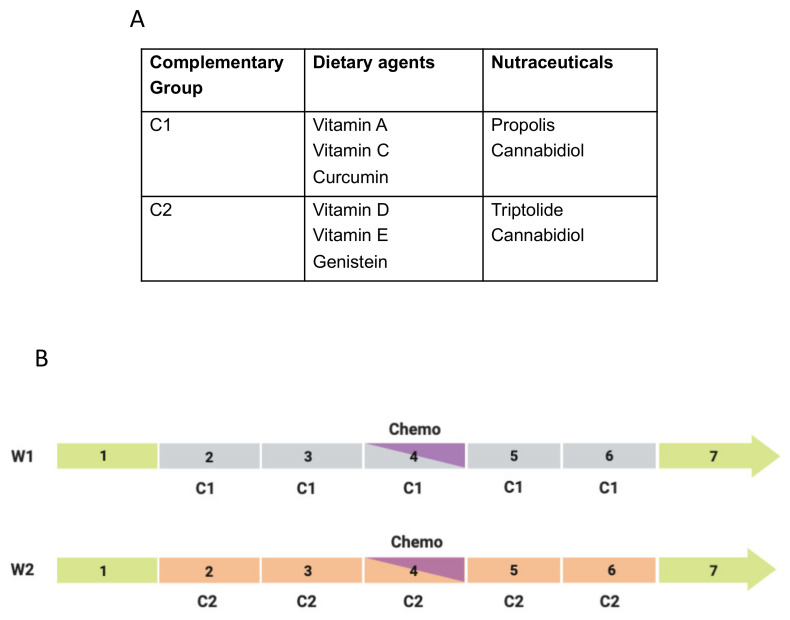
Proposed plan for integrated management of pancreatic cancer (PDAC) patients. (**A**) The 9 complementary agents (“C”) evaluated and adopted in the current review for recommendation for integrative management of PDAC. The agents are divided into two groups, C1 and C2, each comprising 3 dietary and 2 nutraceutical agents, mixed in line with their main modes of action related to the Hallmarks of Cancer. Note that cannabidiol as the most all-round effective of the 3 nutraceutical agents is included in both subgroups, also to even out the numbers. (**B**) Proposed integration scheme based upon weekly cycle of gemcitabine treatment. Colors of days (1–7) denote the following: green (rest), grey and orange (days of consuming the complementary agents, C1 and C2, respectively), purple (chemotherapy on day 4). The integration involving the C1 and C2 subgroups of complementary agents are alternated weekly.

As outlined in Section 2, gemcitabine is most commonly administered in weekly cycles. The day of the chemotherapy is followed by 6 days of ‘rest’, and this is continued for several weeks. This cycle is adopted here as the basis of the integration (Figure 19). 

We have identified 9 compounds with sufficient evidence to be considered as complementary (“C”) to gemcitabine chemotherapy. These include 6 dietary and 3 nutraceutical agents (Section 3.3 and Section 4). The critical question is how can the integration best be achieved? The short answer is ‘we do not know definitively’ since relevant clinical trials are lacking or have only gotten underway recently as the need has become more and more pressing. What we do know is that some 90% of PDAC patients will die within 5 years (70% in the first year) [342]. So, something must be done to improve the survival rate for which we have to be also insightful, if not fully evidence-based. Thus, we propose an integration regime based upon the following logistics:

1. The 9 complementary agents are divided into 2 groups—C1 and C2 (Figure 19A). Each of these contains 5 agents (3 dietary, 2 nutraceutical). One agent (cannabidiol) is in both groups (to even the numbers), chosen due to the increasing all-round evidence in its favor. The mixing took into consideration their main modes of action in relation to the ‘hallmarks of cancer’, e.g., as antioxidant, anti-inflammatory/pro-immune and/or anti-angiogenic [343,344].
2. One group (say C1) is taken for 2 days before the day of the chemotherapy and this is continued on the day of the treatment and for the following two days, making 5 days in total (Figure 19B).
3. Then there are two days of ‘rest’ and then the cycle switches to the C2 group (Figure 19B). This continues as in (2) and then the integration switches back to the C1 agents.
4. These alternating cycles (designed to increase the chance of either group working) continue for the duration of the treatment (ca. 7 weeks).

The proposed scheme is intended for ‘routine’ responders (i.e., those who belong to the 90% who will not survive beyond the 5 years). Accordingly, and since in general, cancer treatment is a dynamic process often needing adjustment, some caveats may be exercised along the way. In particular, ‘poor’ responders (representing the 70% who would be expected die in the first year) may benefit from combined application of all 9 agents throughout the whole treatment period. On the other hand, some (exceptional) patients may prove to be ‘super’ responders who may wish to reduce the quantity of supplementary agents taken, at least temporarily. Interestingly, research is aiming to benefit from such ‘super’ responders by determining their molecular landscape [345]. 

In addition, the following points may need consideration. First, as regards how much of each agent to take, with the limited data available, we suggest simply following the instructions of the manufacturer. Also, (i) advice may be taken from a reputable practitioner of complementary medicine or a dietician, and (ii) dosages may be adjusted depending on the results of the monitoring, again in consultation with a professional. Second, with up to 5 agents to be consumed daily, the question arises as to when to take the various subgroups. Since the general advice is to take supplements with food, we suggest taking one or two supplements each after breakfast, lunch and dinner. Third, routine monitoring of blood levels of the supplementary agents being taken should be done so adjustments can made if necessary (see also Section 7.6). Fourth, we should also note that ‘intermittent dieting’ (i.e., calorie restriction) is increasingly being highlighted as generally beneficial and would be worth considering [116,346]. One possibility would be to exercise this on the days of rest.

## 7. Future Perspectives

PDAC is a widespread disease and is currently almost a ‘death sentence’ for patients, especially if it has already spread when diagnosed. With mortality set to rise over the coming years, more will need to be done to improve patient outlook, both by improving existing treatment methods and discovering novel therapies. As a first step, towards the former, we have used a hierarchical approach to evaluate the effectiveness of a range of complementary agents in potentiating gemcitabine chemotherapy, the most common first-line treatment for PDAC. We should note, however, that some limitations nevertheless would remain. Whilst clinical trials are the ‘gold standard’, final reports were not available in some cases. Nevertheless, we have given positive consideration even to such cases on the basis that sufficient justification must have been provided to run the trial in the first place. Meta-analyses are limited mainly to specific dietary agents. Although in vivo animal models can include molecularly appropriate transgenic models, it is well known that data from animals do not always translate to humans. Finally, since in vitro experiments are relatively plentiful, we have restricted the data to those involving primarily human cells. Nevertheless, it is also well known that results from in vitro experiments may not be reproducible in vivo.

As medical science advances, patients and their supporters will continue scouring the internet both for emerging and existing information. It is important, therefore, to be able to evaluate such ‘information’ properly. In this regard, we would like to suggest that the following three questions are asked, and the answers are adopted as ‘rules’ [21]. (i) Where was the evidence published? Ideally, this should be peer-reviewed and published in an internationally reputable journal. (ii) When was the suggested ‘remedy’ published? Has it stood the test of time? (iii) Has it been replicated by others? Modern scientific discipline demands that solid evidence should be repeatable by others independently.

Some existing issues, which remain to be studied and clarified for improving the outcome of the proposed integrative management of PDAC, are highlighted in the following sections.

### 7.1. Quality Issues

One of the potential problems with dietary supplements and nutraceutical agents is the fact that the marketplace is not strictly regulated so the purity of the products can be questionable. Bad quality can even result in tissue damage and adversely affect clinical outcome. For example, a study on milk thistle reported high concentrations of mycotoxins and several pesticides, as well as substantial presence of microbiological contamination [347]. We should also note that dietary and nutraceutical agents will often be taken as the original product, the precise composition of which may be variable and depend on source, e.g., soil in which these are grown [348]. It is important, therefore, that all supplementary agents to be consumed are obtained strictly from reputable sources and ideally carry the Traditional Herbal Registration (THR) Certification Mark.

### 7.2. Emerging Modalities

Medical research is advancing fast and so is our understanding of the potential usefulness of dietary and nutraceutical agents in cancer, including PDAC, prevention and management. In addition to the 9 agents adopted here, there are the following dietary and nutraceutical compounds for which no clinical trials have been reported but there is promising in vivo and in vitro evidence (including from combination treatments): Vitamin B9, sulforaphane, epigallocatechin. Finally, for the following, currently there are comparable data but only from in vitro experiments: Capsaicin, resveratrol, artemisinin, garcinol, thymoquinone and emodin. In time, evidence may increase for these to make them acceptable for integration. Indeed, significant further progress can be expected in this field [349]. Furthermore, recently developed elaborate computer algorithms (e.g., VIPER) could identify viable novel targets [https://www.sciencemag.org/news/2020/06/computer–algorithms–find–tumors–molecular–weak–spots]. In considering the possibility of adopting multiple complementary agents, attention should also be given to the avoidance of any antagonism [350]. 

### 7.3. Molecular Mechanisms

Unfortunately, our understanding of the molecular mechanisms associated with the range of complementary agents covered here is extremely limited both individually and in combination with gemcitabine. We have given an overview for the individual agents adopted in Table 2 and Table 3. Basically, the difficulty is not surprising since most dietary and nutraceutical natural agents have multiple modes of action. For example, even a common spice like curcumin has anti-inflammatory, monoaminergic, antioxidant, immune-modulating and neuroprotective effects, with several different molecular mechanisms underlying each [351]. In the context of ‘combination therapy’, many unknowns remain as regards temporal (sequential) effects and combination ratios (and doses) in order to maximize synergies and minimize/eliminate antagonistic effects. At the gene level, a potentially important concept in combinations is “synthetic lethality”, an interaction involving two genes when the perturbation of either gene alone is viable, but the perturbation of both genes simultaneously results in loss of viability [352]. Thus, identification and molecular characterization of robust synthetic lethal genetic interactions could enable exploitation of synthetic lethality in cancer treatment. Advances in techniques like next-generation sequencing, coupled with model organisms, tumor genomes and human cell lines, promise to enable this. In conclusion, much more work is required to elucidate in depth the modes of action of complementary factors in order to maximize the impact of integrated management of PDAC.

### 7.4. Precision Medicine

This is the age of ‘precision medicine’ (what used to be called more commonly “personalized medication”). Everything that we have said and advocated so far, although evidence-based, will to some extent have a personalized basis [353]. Accordingly, one would expect the impact of the kind of measures highlighted here to vary from person to person and the molecular prolife of PDAC [9,354,355,356]. Consequently, there will be some degree of ‘hit-and-miss’, which patients (and professionals) will need to experiment with. If available and affordable, a professional dietician with expertise in the cancer area could also help improve the patient survival rate [357]. Thus, integrated management may work better on some patients than others. Furthermore, understanding the genetic differences amongst patients and their cancers can also ultimately lead to markedly improved outcomes [358,359]. An interesting development in this area is the wearable technology “DnaNudge” which can optimize the dietary choices of individuals [https://www.dnanudge.com/].

### 7.5. Monitoring

Cancer, including PDAC, is an incredibly complex process. Cancer cells can change their character dynamically in space and time and in response to treatment. Furthermore, response to diet and its impact on disease parameters can be highly personalized, as highlighted in the previous section [360]. All these necessitate constant monitoring of the integrated management, as regards both clinical parameters and complementary applications. Regarding the latter, for example, the vitamin D level should be checked before every chemotherapy session and once a month after treatment has ended. Also, since liver has a special place in PDAC, a ‘liver function test’ may be carried out similarly. Both these tests are readily possible, and their frequency can be adjusted depending on progress. Interestingly, a recent study has shown that metabolic profiling of urine can be used to determine the effectiveness of different diets on individuals, thereby raising the concept of ‘precision nutrition’ [361]. An important development in monitoring of nutritional or disease status is use of wearable devices [362]. A flexible ‘tattoo’ patch was recently shown to be capable of measuring vitamin C levels in sweat as means of tracking the body’s vitamin C status to facilitate any necessary correction of nutritional deficiencies [363]. 

### 7.6. Role of the Brain

Recent evidence suggests that ‘optimism’ can significantly boost general physical and mental health [364]. The fact that tumors, including those of pancreas, are frequently innervated and the nerve input plays a significant role in tumor progression provide a mechanistic basis for the modulatory role of mental state in cancer [365,366,367]. Furthermore, immune tissues are also innervated, and cells of the immune system possess neurotransmitter receptors [368]. Indeed, a remarkable experimental study showed that stimulation of the ventral tegmental area (VTA) of the brain would suppress the growth of human lung cancer and melanoma implanted in mouse models by 40–50%, possibly by boosting anti-tumor immunity [369]. VTA is a part of the brain’s ‘reward system’ and uses dopamine as its primary messenger. In fact, a range of neurotransmitters and ion channel modulators also exert significant influence upon the cancer process, from initiation to metastasis, and would be worthy of further investigation [370,371,372]. A major advantage here is the availability of an arsenal of clinically viable drugs targeting ion channels and neurotransmitter receptors that can be repurposed against cancer [373]. 

### 7.7. Clinical Trials

Here, all the dietary and nutraceutical agents that we adopted for ‘integration’ have been justifiably subjected to some form of clinical trial, our primary criterion. There is great need to carry our more trials on the wide range of promising agents that exist, also to resolve the kinds of speculations that continue in what is sometimes called the field of “complementary and alternative medicine” (CAM). Furthermore, some agents may appear to have both anti- and pro-cancer properties. For example, the evidence presented in Section 3.2.6 clearly suggested that coffee has anti-cancer properties. However, coffee is also well known to be acidic which promotes cancer, including PDAC (Section 3.1.1). This apparent contrast has two important implications. First, it is the net homeostatic effect of a given dietary (or nutraceutical) agent that will determine whether it would inhibit or promote cancer. Second, clinical trials involving combination treatments are ultimately necessary in order to reach a firm conclusion and best decide about integration.

## 8. Overall Conclusions

We aimed here to evaluate and use evidence-based medicine and science with mechanistic insights to demonstrate how integrated approaches can be used to maximize the chance of surviving PDAC or at least extending survival times whilst maintaining quality of life. In overall conclusion, first, we believe that integrated management of PDAC is strongly warranted and should be supported further by clinical trials. Second, the available evidence supports the hypothesis that integrated management will currently provide the best outcome for patients. With the ongoing advances in the field, therefore, the future should be bright for PDAC patients, cancer patients and those prone to cancer generally, as well as their carers.

## Figures and Tables

**Table 1:** Clinical trials involving some of the complementary agents adopted in this review, in part combined with chemotherapy, against pancreatic cancer. The list is in order of appearance in the text (indicated by serial letters). For a few of these, the reference number and/or the completion date could not be deciphered. The last column gives the publication resulting from the trial, if any. NRP indicates ‘no results posted’ (as stated on the official trial site). In several cases, no publication appeared (yet) to have followed. The comment in (F) was apparent in the limited information given on the trial site.

Serial Letter	National Clinical Trial (NCT) Number	Title	Primary Investigator	Site(s)	No. of Patients	Completion Date	Reference(s)/Comments
A	01019382	Phase II trial of the effect of gemcitabine with intravenous omega-3 fish oil infusion in patients with unresectable pancreatic adenocarcinoma	Ashley Dennison	Leicester University Hospitals (UK)	50	June 2014	[33]
B	03307148	A Phase 1b study repurposing ATRA as stromal targeting agent along with gemcitabine and nab-paclitaxel for pancreatic cancer (STAR-PAC)	David Propper	Barts and The London NHS Trust (UK)	34	March 2018	No results posted (NRP)
C	01852890	Gemcitabine, ascorbate, radiation therapy for pancreatic cancer, Phase I	Joseph J Cullen	The University of Iowa Hospitals & Clinics (USA)	16	January 2019	[34]
D	02905578	A Phase II trial of pharmacological ascorbate, gemcitabine, and nab-paclitaxel for metastatic pancreatic cancer (PACMAN 2.1)	Joseph J Cullen	University of Iowa (USA)	65	December 2025	-
E	03410030	Phase Ib/II trial of high dose ascorbic acid (AA) + nanoparticle paclitaxel protein bound + cisplatin + gemcitabine (AA NABPLAGEM) in patients who have no prior therapy for their metastatic pancreatic cancer	Gayle S Jameson	HonorHealth Research Institute (USA)	36	July 2020	-
F	02896907	A pilot study of intravenous ascorbic acid and FOLFIRINOX in the treatment of advanced pancreatic cancer	James Posey	Sidney Kimmel Cancer Center at Thomas Jefferson University (USA)	8	October 2019	Some adverse effects reported, reason(s) and any benefit not clear.
G	03520790	Vitamin D receptor agonist paricalcitol plus gemcitabine and nab-paclitaxel in patients with metastatic pancreatic cancer	Kimberly Perez	Dana-Farber Cancer Institute (USA)	112	November 2025	-
H	03519308	A pilot study of perioperative nivolumab and paricalcitol to target the microenvironment in resectable pancreatic cancer	Peter O’Dwyer	Abramson Cancer Center of the University of Pennsylvania (USA)	20	July 2022	-
I	03415854	A Phase II pilot trial of paclitaxel protein bound plus cisplatin plus gemcitabine and the addition of paricalcitol upon disease progression in patients with previously untreated metastatic pancreatic ductal adenocarcinoma (NABPLAGEMD)	Erkut Borazanci	HonorHealth Research Institute (USA)	14	December 2020	-
J	03300921	A Phase Ib pharmacodynamic study of neoadjuvant paricalcitol in resectable pancreatic cancer	Peter O’Dwyer	Abramson Cancer Center of the University of Pennsylvania (USA)	20	April 2020	Suspended/NRP
K	03331562	A SU2C Catalyst ^®^ randomized Phase II trial of the PD1 inhibitor pembrolizumab with or without a vitamin D receptor agonist paricalcitol in patients with stage IV pancreatic cancer who have been placed in best possible response	Daniel Von Hoff	Translational Genomics Research Institute (USA)	24	June 2021	-
L	00985777	A Phase I dose-escalation study of the safety, pharmacokinetics, and pharmacodynamics of vitamin E δ-tocotrienol administered to subjects with resectable pancreatic exocrine neoplasia	Gregory Springett	H. Lee Moffitt Cancer Center and Research Institute (USA)	26	June 2015	[35]
M	-	A Phase I/II study of gemcitabine-based chemotherapy plus curcumin for patients with gemcitabine-resistant pancreatic cancer	Masashi Kanai	Kyoto University Hospital (Japan)	21	-	[36]
N	01182246	Safety, pharmacokinetics and efficacy of AXP107-11 in combination with standard gemcitabine (Gemzar^®^) treatment in patients with locally advanced or metastatic, unresectable, adenocarcinoma of the pancreas, stage III-IV: a prospective, open label, multi-center, sequential Phase Ib/IIa study	Mattias Löhr	Karolinska Institute (Sweden)	44	March 2016	[37]
O	01375088	Phase I assessing the preventing and therapeutic effect of propolis in radiotherapy induced mucositis of head and neck cancers	Arghavan Tonkaboni	Mashhad University of Medical Sciences (Iran)	20	March 2011	NRP
P	03416127	Effect of propolis or metformin administration on glycemic control in patients with type 2 diabetes mellitus without pharmacological treatment	Manuel González Ortiz	Intstituto de Terapeútica Experimental y Clínica, Universidad de Guadalajara (Mexico)	36	October 2020	-
Q	03117920	MinPAC: A Phase II, international open label trial of Minnelide™ in patients with refractory pancreatic cancer	David Propper	Barts & The London NHS Trust (UK)	35	May 2020	NRP
R	03129139	A Phase I, multi-center, open-label, dose-escalation, safety, pharmacokinetic, and pharmacodynamic study of Minnelide™ capsules given alone or in combination with protein-bound paclitaxel in patients with advanced solid tumors	Cameron Wright	Minneamrita Therapeutics LLC (USA)	54	December 2021	-
S	03245658	The effect of medical cannabis inpatients with palliative pancreatic cancer	Jens Rikardt Andersen	University of Copenhagen (Denmark)	32	October 2018	NRP
T	03984214	Efficacy and safety of dronabinol in the improvement of chemotherapy-induced and tumor-related symptoms in advanced pancreatic cancer	Felix Keil	Arbeitsgemeinschaft Medikamentoese Tumortherapie (Austria)	140	June 2022	-
U	02295956	Preoperative rehabilitation during neoadjuvant therapy for pancreatic cancer: A pilot study	Matthew H. Katz	Anderson Cancer Center (USA)	75	June 2019	[38]

**Table 2:** Dietary agents adopted in this review for the integrated management of pancreatic cancer (PDAC). For each agent, chemical formulae, main modes of action (cellular effects) and the natural sources of individual agents are given. For further details, see main text.

Agent	Formula	Mechanisms of Action/Cellular Effects	Food Sources
Vitamin A (Retinol)	C_20_H_30_O	Multiple—metabolic substrate, transcriptional regulator of growth and differentiation (epithelial, bone and immune cells)	Carrots, eggs, leafy green vegetables, carrots, dark leafy green vegetables, dried apricots, cantaloupe, bell peppers, fish, liver, tropical fruits
Vitamin C	C_6_H_8_O_6_	Regulator of oxidation-reduction processes, mainly as an antioxidant and immune regulation	Citrus fruits, broccoli, cantaloupe, cauliflower, kale, kiwi, papaya, sweet peppers, tomatoes
Vitamin D: D2 (Ergocalciferol) D3 (Cholecalciferol)	C_28_H_44_O C_27_H_44_O	Multiple—wide-ranging genomic and non-genomic regulator, including of calcium metabolism and immune response	Oily fish, eggs, mushrooms
Vitamin E	C_29_H_50_O_2_	Mainly an antioxidant that inhibits production of reactive oxygen species	Nuts, seeds, sunflower oil, soybeans, avocado, green leafy vegetables, spinach
Curcumin	C_21_H_20_O_6_	Pleiotropic—several modes of action especially antioxidant and anti-inflammatory	Turmeric
Genistein	C_15_H_10_O_5_	Multiple—modulator of growth factor signaling, pro-apoptotic, inhibitor of cancer cell survival, proliferation, angiogenesis	Soybeans and soy products

**Table 3:** Nutraceutical agents adopted in this review for the integrated management of pancreatic cancer (PDAC). For each agent, chemical formulae, main modes of action (cellular effects) and the natural sources of individual agents are given. For further details, see main text.

Agent	Formula	Mechanisms of Action/Cellular Effects	Source
Propolis	Variable	Multiple—anti-inflammatory, antioxidant	Honey (comb)
Triptolide	C_20_H_24_O_6_	Complex—anti-inflammatory, antioxidant, immune-modulator	Thunder god vine
Cannabidiol	C_21_H_30_O_2_	Multi-modal—immune-modulator, neuromodulator (analgesic, anti-convulsant)	Cannabis

## Abbreviations and Glossary

This is included so non-experts (especially patients) can have a basic understanding of some of the scientific/medical terminology used in this review.

Adjuvant	supportive therapy applied after initial main treatment.
Akt	(protein kinase B), an enzyme that plays a role in cell metabolism, especially the insulin pathway.
AMPK	(5′ adenosine monophosphate-activated protein kinase), an enzyme involved in cell metabolism.
Analogue	a compound similar in structure to another.
Angiogenesis	formation of new blood vessels.
Apoptosis	death of cells by a pre-programmed genetic mechanism.
Autophagy	a mode of cell death, removing damaged proteins, organelles and pathogens.
Beta (β)-catenin	protein that plays a role in cell signaling and cell-cell adhesion.
Bioavailability	the proportion of a substance that can be absorbed by the body.
Biomarker	a molecule from which a biological process can be identified.
BMI	(body-mass index, the body weight in kg divided by the square of the body height in meters), a measure of obesity.
Cachexia	late-stage weakness and wasting of the body due to severe illness.
CA9	(carbonic anhydrase-9), an enzyme involved in controlling the body’s acidity.
Case-control study	a retrospective study comparing the effect of a given measurable factor on a group of people, compared with a control (non-exposed) group.
Carcinogenesis	formation of cancer.
Caspase-3	an enzyme involved in apoptosis.
Clinical trial	testing of a drug on humans prior to official approval, beginning with basic toxicity (phase I) and leading to more detailed evaluation on increasing numbers of patients (phases II–IV).
Cohort study	a longitudinal (time-course) study testing the effect of a certain treatment on a group of people, normally in comparison with a control (untreated) group.
Cytokine	signaling molecule (protein) involved in cell signaling, common in the immune system.
Desmoplasia	dense fibrous tissue.
EGF	(epidermal growth factor), a protein involved in the growth, proliferation and differentiation of cells.
EGFR	(epidermal growth factor receptor), a protein acted upon by epidermal growth factor.
EMT	(epithelial-mesenchymal transition), an early event/morphological change in cancer cells becoming invasive.
Epigenetic	regulation of gene expression levels.
Flavonoid	groups of chemicals, pigments, found in plants that have anti-oxidative and anti-inflammatory properties.
5-FU	(5-fluorouracil), a common anti-cancer drug (‘chemotherapy’) often used in combination with other medications.
FOLFIRINOX	(folinic acid, fluorouracil, irinotecan and oxaliplatin), a combination chemotherapy drug.
GPCR	(G protein-coupled receptor), a protein found on the cell membrane that interacts with incoming signaling molecules.
Hedgehog	a signaling mechanism operating in cells, especially during development, and tumorigenesis; sonic hedgehog is a main protein.
Hydrogel	cross-linked polymer gel that can absorb and retain water.
Hyperplasia	increase in the number of cells within an organ or tissue, forming a barrier.
IGF	(insulin-like growth factor), a protein involved in the growth, proliferation and differentiation of cells; shares the same receptor with insulin.
Intraperitoneal	an injection into the abdominal cavity of the body.
Isoform	a protein with the same function as another but differing slightly in structure.
Isomers	a molecule with the same formula but different structure.
KPC	a genetically modified mouse model of pancreatic cancer.
KRAS	a gene that, when mutated, can cause cells to become cancerous.
Macrophage	a type of cell found in the immune system, detects and destroys harmful microorganisms.
MAPK	(mitogen associated protein kinase), an enzyme that has a central involvement in the cancer process.
Meta-analysis	quantitative study that uses the results of multiple previous analyses to achieve a consensus opinion.
Microbiome	microorganisms, especially bacteria, that reside in parts of the body e.g., the gut.
Neo-adjuvant	initial therapy applied before the main treatment, e.g., chemotherapy or radiation therapy.
Neoplasm	new and abnormal growth of tissue, commonly leading to cancer.
NF-kB	(nuclear factor kappa-light-chain-enhancer of activated B cells), a protein involved in gene expression and patho/physiological regulation.
Notch	a protein involved in cellular signaling, especially during development, and cancer.
Orthotopic	something occurring in its normal location.
PanIN	(pancreatic intraepithelial neoplasia), a pathological indicator of pancreatic cancer and its grade.
PDAC	(pancreatic ductal adenocarcinoma), the most common type of pancreatic cancer.
PDX	(patient derived xenograft), human cancer tissue implanted and surviving in an animal model.
PI3K	(phosphoinositide 3-kinase), an enzyme involved in cell signaling.
Polyphenol	a type of plant-derived chemical with health benefits.
Rho-kinase	a protein/enzyme involved in cell signaling, often as an intermediary.
STAT3	(signal transducer and activator of transcription 3), a protein involved in cell signaling and gene regulation.
T-cell	a type of lymphocyte (white blood cell) that is a central part of the immune system.
TGFβ	(transforming growth factor beta), a primary signaling protein involved in cellular mechanisms.
TRAIL	(tumor necrosis factor-related apoptosis-inducing ligand), a protein that promotes programmed cell death.
Transgenic	an organism that contains genetic information from another organism, often used as cancer models.
Wnt	(Wingless and Int-1), a signaling mechanism operating in cells, especially during development and tumorigenesis; incorporates a variety of Wnt proteins.
Xenograft	a tissue from one species, grafted experimentally to another (often human to mouse).
